# Transcriptomic and Proteomic Insights into Mucosal Immune Responses of Asian Seabass (*Lates calcarifer*) After Sequential Mucosal Vaccination Against Bacterial Pathogens

**DOI:** 10.3390/ijms27177652

**Published:** 2026-08-26

**Authors:** Chatchai Rodwihok, Kim D. Thompson, Pakapon Meachasompop, Benchawan Kumwan, Yosapon Adisornprasert, Pimrawee Chaemlek, Prapansak Srisapoome, Patcharapong Thangsunan, Pattanapong Thangsunan, Wararut Buncharoen, Passakorn Kingwascharapong, Channarong Rodkhum, Natthapong Paankhao, Anurak Uchuwittayakul

**Affiliations:** 1 Special Research Incubator Unit for Development and Application of Vaccine Delivery Systems for Aquatic Animals, Department of Aquaculture, Faculty of Fisheries, Kasetsart University, Bangkok 10900, Thailand; 2 Laboratory of Aquatic Animal Health Management, Department of Aquaculture, Faculty of Fisheries, Kasetsart University, Bangkok 10900, Thailand; 3 Center of Excellence in Aquatic Animal Health Management, Faculty of Fisheries, Kasetsart University, Bangkok 10900, Thailand; 4 Moredun Research Institute, Pentlands Science Park, Penicuik EH26 0PZ, UK; 5 Division of Biochemistry and Biochemical Innovation, Department of Chemistry, Faculty of Science, Chiang Mai University, Chiang Mai 50200, Thailand; 6 Office of Research Administration, Chiang Mai University, Chiang Mai 50200, Thailand; 7 Center of Excellence for Innovation in Chemistry, and Research Laboratory on Advanced Materials for Sensor and Biosensor Innovation, Material Science Research Center, Faculty of Science, Chiang Mai University, Chiang Mai 50200, Thailand; 8 Department of Biology, Faculty of Science, Chiang Mai University, Chiang Mai 50200, Thailand; 9 Department of Fishery Products, Faculty of Fisheries, Kasetsart University, Bangkok 10900, Thailand; 10 Center of Excellence in Fish Infectious Diseases (CE FID), Faculty of Veterinary Science, Chulalongkorn University, Bangkok 10330, Thailand; 11 Kamphaeng Saen Fisheries Research Station, Faculty of Fisheries, Kasetsart University, Kamphaeng Saen Campus, Nakhon Pathom 73140, Thailand

**Keywords:** Asian seabass, mucosal immunity, nanoemulsion vaccine, oral hydrogel vaccine, transcriptomics, proteomics

## Abstract

Bacterial diseases caused by *Flavobacterium covae* (*Fc*), *Vibrio harveyi* (*Vh*), *Vibrio vulnificus* (*Vv*) and *Photobacterium damselae* (*Pd*) seriously constrain Asian seabass aquaculture. Here we dissect the mucosal immune mechanisms engaged by a five-month sequential vaccination strategy that combines nanoemulsion immersion priming with multivalent oral hydrogel boosting. Juvenile seabass were vaccinated, then challenged with *F. covae* by freshwater immersion and with a *Vibrio*–*Photobacterium* (*Vh/Vv/Pd*) mix by immersion or intraperitoneal injection. Gills were sampled after immersion challenges and intestine after injection, and profiled by RNA sequencing and label-free quantitative proteomics, with selected genes validated by RT-qPCR. Principal component analysis showed clear separation of vaccinated and control fish in all tissues and challenges, indicating a strong and coherent transcriptional reprogramming. Vaccination markedly increased the number of upregulated genes, with Gene Ontology enrichment revealing dominant signatures of ribosome biogenesis, RNA processing, lysosomal organization and immune response. KEGG analysis highlighted cytokine receptor interaction; NOD and Toll-like receptor signaling; oxidative phosphorylation; and phagosome, lysosome and cell adhesion molecule pathways, consistent with heightened antimicrobial readiness. Volcano plots and focused heatmaps showed strong induction of interferon-stimulated genes, cytokines and chemokine receptors, complement components, macrophage mannose receptor, epithelial barrier mediators and numerous immunoglobulin transcripts, with tissue- and challenge-specific patterns. Proteomics corroborated these trends, demonstrating a higher abundance of immunoglobulin heavy chains, complement proteins, cathepsins, heat shock and redox chaperones, ribosomal proteins and cytoskeletal and adhesion regulators in vaccinated mucosae. Integrated pathway mapping linked endothelial adhesion molecules and leukocyte integrins with T cell costimulation networks and an intestinal immune network for immunoglobulin production, including enhanced pIgR-mediated transcytosis. Overall, the sequential vaccination regimen was associated with coordinated transcriptomic and proteomic signatures related to epithelial responses, innate immunity, and humoral immune functions across gill and intestinal tissues. These molecular patterns were accompanied by improved survival following bacterial challenge; however, the present data do not directly demonstrate the functional activity of the inferred immune mechanisms in Asian seabass.

## 1. Introduction

Antibiotics remain widely used for outbreak control [1,2,3], but their reliance accelerates antimicrobial resistance and raises environmental and food safety concerns [4,5]. Vaccination offers a sustainable alternative [6,7]; yet injectable formats are impractical for high-throughput juvenile handling and can induce stress-related losses [8,9,10]. Mucosal immunization by immersion and oral routes aligns better with commercial realities, enabling mass administration while directly targeting the surfaces where most aquatic pathogens first interact with the host [11,12].

Despite this promise, conventional mucosal vaccines often yield modest or short-lived protection. Antigens delivered by immersion may have limited residence time on epithelial surfaces, whereas orally administered antigens can be degraded before reaching inductive sites [12,13]. Delivery technologies have therefore been developed to overcome these limitations. Nanoemulsions can improve antigen stability, epithelial adhesion, and uptake during immersion [14,15,16], while biodegradable hydrogels can protect antigens during gastrointestinal transit and provide controlled release that prolongs exposure to gut-associated lymphoid tissues [17,18,19]. In parallel, fish vaccination strategies have progressed from monovalent formulations targeting a single pathogen toward bivalent and multivalent approaches intended to broaden protective coverage in production systems where several bacterial diseases may occur concurrently. In Asian seabass, monovalent immersion-prime and oral-boost vaccination against *V. harveyi* has been shown to induce specific immune responses and protection, while similar immersion and oral nanoemulsion approaches have been investigated against *V. vulnificus* [20,21,22]. Bivalent nanovaccine strategies have also been developed to simultaneously target streptococcosis and columnaris disease [21], and a hydrogel-based multivalent vaccine containing multiple *Vibrio* antigens has been shown to enhance immune responses and protection against vibriosis [19]. Compared with monovalent vaccines, which provide focused antigenic stimulation against a specific pathogen, multivalent formulations offer the potential advantage of broader coverage against multiple bacterial threats within a single vaccination program. However, increasing antigenic complexity may also influence antigen presentation, immune competition, and the magnitude of responses to individual components. Sequential prime–boost schedules that combine complementary delivery platforms and antigen combinations may therefore provide a practical strategy to enhance the magnitude and durability of mucosal immune responses across multiple compartments [20,21,22].

Teleost mucosal immunity is organized within the skin-, gill-, and gut-associated lymphoid tissues, which integrate innate recognition, antigen presentation, cytokine orchestration, and B-cell responses, including IgM, IgD, and IgT homologs at barrier surfaces [12,23,24,25]. Emerging work suggests that stimulation at one mucosal site can shape responses at others through trafficking and shared cytokine networks [6,26,27]. Systematically defining how a nanoemulsion immersion prime followed by an oral hydrogel boost remodels these pathways across gill, skin, and intestine is therefore central to rational vaccine design for seabass [20,22,28].

Transcriptomics was included to define the breadth of vaccination-associated regulatory responses at the gene-expression level, including upstream signaling programs and low-abundance regulatory transcripts that may not be consistently detected by shotgun proteomics, and to support pathway-level analysis of the mucosal response [29,30,31]. Proteomics was used for a different purpose: to determine which components of these transcriptional programs were also represented by measurable changes in protein abundance. Thus, the two approaches addressed distinct biological questions. RNA-seq mapped broad regulatory programs and candidate pathways, whereas proteomics assessed the protein-abundance layer of selected processes [28,32]. Because mRNA abundance and protein accumulation can diverge because of post-transcriptional regulation, translation efficiency, protein stability, turnover, and temporal delay, we did not assume one-to-one transcript–protein correspondence. Formal investigation of discordant transcript–protein pairs and their regulatory mechanisms would require additional time-resolved and targeted experiments and was beyond the scope of the present study [33,34,35].

The Asian seabass (*Lates calcarifer*) is a cornerstone of Indo-Pacific aquaculture because of its rapid growth, euryhaline physiology, and strong consumer demand [36,37,38]. With ongoing intensification, however, bacterial diseases increasingly disrupt productivity and profitability. In Thailand, four pathogens are consistently implicated across production systems: *Flavobacterium covae*, *Vibrio harveyi*, *Vibrio vulnificus*, and *Photobacterium damselae*. While *F. covae* is commonly associated with freshwater operations, *V. harveyi*, *V. vulnificus*, and *P. damselae* predominate in brackish to marine environments, highlighting the ecological breadth of risk and the need for cross-context protection [19,39,40,41,42,43,44].

Here we investigate a five-month sequential mucosal vaccination strategy for Asian seabass that uses nanoemulsion-based immersion priming and hydrogel-based oral boosting against these four pathogens [19,21]. Transcriptomic profiling was used to map broad vaccination-associated gene-expression programs and enriched pathways in gill and intestinal tissues following pathogen exposure [28,30]. In parallel, label-free quantitative proteomics was used to determine which selected immune and cellular processes were also represented by changes in protein abundance [32,34,35]. This design was intended to compare regulatory and protein-abundance layers at the level of biological processes rather than to assume direct transcript–protein equivalence. We hypothesized that the sequential regimen would be associated with molecular signatures related to epithelial, innate, and adaptive immune responses across mucosa-associated lymphoid tissues [6,26,27].

Our objectives were to: (i) delineate tissue-specific immune pathways associated with the sequential nanoemulsion plus hydrogel regimen, (ii) characterize broad transcriptional programs and determine which selected functional processes were also represented at the protein-abundance level, and (iii) relate these molecular signatures to vaccine-associated improvements in host protection. By combining systems-level transcriptomics and proteomics with a practical, noninvasive vaccination schedule, this study provides a molecular framework for further investigation of mucosal vaccine responses in Asian seabass.

## 2. Results

### 2.1. Sequencing Data and Quality Summary

Across 18 libraries, sequencing data quality was uniformly high. Median clean read depth was 23.3 million reads (range 21.7–24.6 million) with a median clean base yield of 6.95 Gb (6.49–7.35 Gb). Base composition was stable, with GC at a median of 47.5% and tight dispersion. Ambiguous base content was consistently low at 0.01% for all samples. Base-calling quality was strong, with Q20 at a median of 98.58% (98.47–98.66%) and Q30 at a median of 94.67% (94.12–94.89%). No samples breached practical QC thresholds for Q30 < 85%, Q20 < 95%, or N% > 1.0%. Collectively, these metrics indicate stable library preparation and sequencing, with no evidence of batch effects or quality–depth trade-offs (Table S2).

### 2.2. Global Expression Patterns

**Figure 1:**
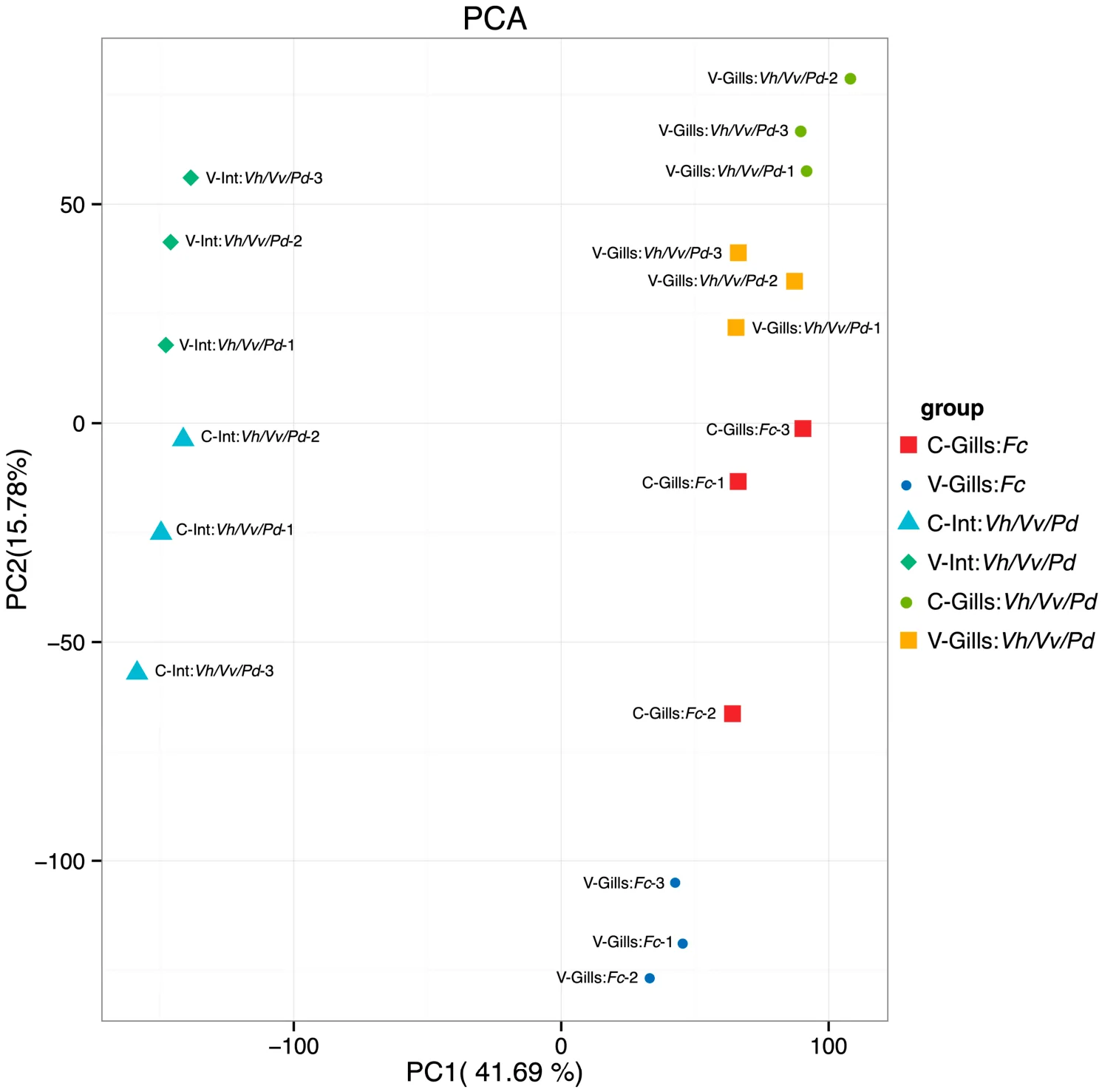
Principal component analysis (PCA) of RNA-seq profiles from gills and intestine of control and vaccinated Asian seabass (*Lates calcarifer*) following bacterial challenge. Each point represents one biological replicate (*n* = 3 per group) and is labeled as C (control) or V (vaccinated), followed by tissue and challenge type: C-Gills:Fc and V-Gills:Fc (gills after *F. covae* immersion), C-Gills:*Vh/Vv/Pd* and V-Gills:*Vh/Vv/Pd* (gills after *V. harveyi*/*V. vulnificus*/*P. damselae* immersion), and C-Int:*Vh/Vv/Pd* and V-Int:*Vh/Vv/Pd* (intestine after *Vh/Vv/Pd* injection).

Principal component analysis (PCA) showed clear separation by treatment and tissue. Vaccinated gills and intestine clustered apart from their respective controls, with tight grouping of biological replicates. PC1 explained 41.69 percent of the variance and captured the primary vaccination effect, while PC2 (15.78 percent) reflected tissue and challenge context. In gills exposed to *F. covae* and in gills and intestine challenged with the mixed pathogen including *V. harveyi/V. vulnificus/P. damselae*, vaccinated samples consistently shifted in the same direction along PC1, indicating a strong and coherent transcriptional reprogramming relative to controls. These patterns support a vaccine-driven modulation of mucosal responses across tissues and pathogen exposures (Figure 1).

### 2.3. Differentially Expressed Genes and Enriched Pathways

#### 2.3.1. Biological Process

**Figure 2:**
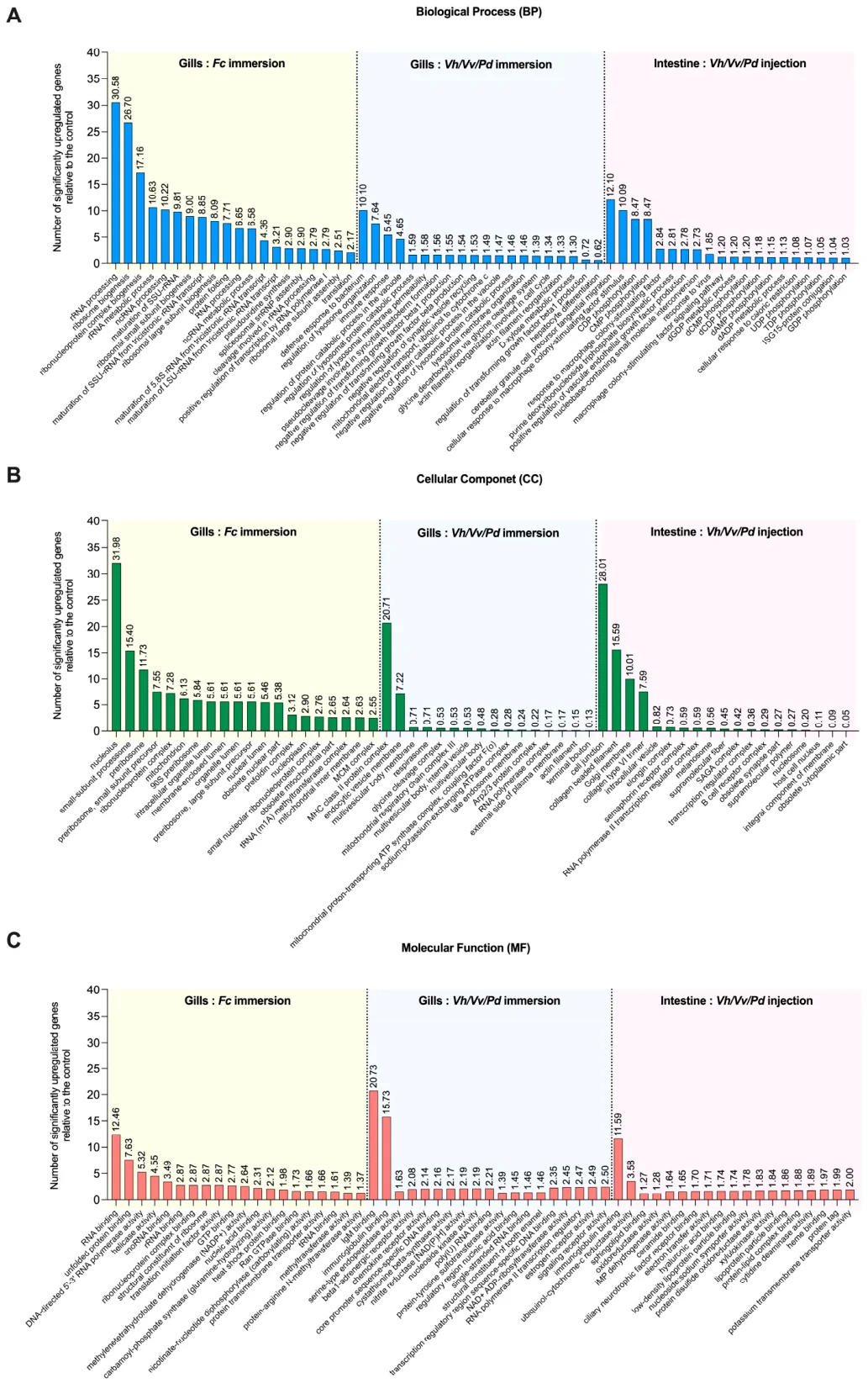
Gene Ontology (GO) enrichment of significantly upregulated genes in gills and intestine of vaccinated Asian seabass relative to control fish after bacterial challenge. (**A**) shows enriched Biological Process (BP) terms, (**B**) shows Cellular Component (CC) terms, and panel (**C**) shows Molecular Function (MF) terms.

Across tissues and challenges, upregulated genes converged on RNA handling, immune–lysosomal activity, and nucleotide metabolism. In gills after *Fc* immersion, top terms were rRNA processing, ribosome biogenesis, ribonucleoprotein complex biogenesis, and ncRNA processing, together with immune response, defense to bacterium, regulation of lysosome organization, and deoxynucleotide phosphorylation including ISG15-protein conjugation. In gills after *Vh/Vv/Pd* immersion, ribosome and RNA biogenesis remained dominant with translation and positive regulation of RNA polymerase I, alongside defense response to bacterium, regulation of lysosome organization and membrane permeability, and mitochondrial electron transport from ubiquinol to cytochrome c. In intestine after *Vh/Vv/Pd* injection, leading terms again centered on translational machinery (rRNA processing, ribosome biogenesis, ncRNA and rRNA metabolic processes) with immune response and regulation of lysosomal protein catabolism, plus high-ranking nucleotide and small-molecule interconversion pathways (Figure 2A).

#### 2.3.2. Cellular Component

In gills after *Fc* immersion, enrichments localized to nuclear ribosome-biogenesis hubs, notably the nucleolus, small-subunit processome, and preribosome, with ribonucleoprotein complexes and the organelle/nuclear lumen; mitochondrial inner membrane and respiratory assemblies were also represented, and vesicle/endosomal membranes appeared as secondary signals. In gills after *Vh/Vv/Pd* immersion, the nucleolus, SSU processome, 90S preribosome, and preribosome again dominated, extending to ribonucleoprotein complexes and nucleoplasm, with clearer endomembrane trafficking signatures (endocytic vesicle membrane, multivesicular body and internal vesicles) and mitochondrial complexes including the respirasome and respiratory chain complex III. In intestine after *Vh/Vv/Pd* injection, top terms clustered at the nucleolus, preribosome, SSU processome, and nuclear or organellar lumen, with pronounced mitochondrial features (mitochondrial inner membrane, ATP synthase F(o), respirasome); lower-magnitude signals included RNA polymerase complexes, Arp2/3 and actin filament components, and immune-linked complexes such as MHC class II and B-cell receptor (Figure 2B).

#### 2.3.3. Molecular Function

In gills after *Fc* immersion, the core program centered on RNA binding, rRNA binding, ribonucleoprotein-complex binding, the structural constituent of ribosome, and supporting helicase and translation initiation factor activities, together with GTP binding and broad nucleic acid binding. In gills after *Vh/Vv/Pd* immersion, a similar RNA–ribosome axis predominated, with added immune and metabolic features including immunoglobulin or IgM binding, serine-type endopeptidase activity, chemokine receptor and signaling receptor activities, and redox terms such as ubiquinol–cytochrome-c reductase and general oxidoreductase activity. In intestine after *Vh/Vv/Pd* injection, translational functions remained prominent (RNA binding, rRNA binding, structural constituent of ribosome, translation initiation factor activity), accompanied by energy and nucleotide handling functions including electron-transfer and oxidoreductase activities, IMP dehydrogenase activity, protein disulfide oxidoreductase activity, and nucleoside kinase activity (Figure 2C).

### 2.4. KEGG Pathway Enrichment of Upregulated Genes

**Figure 3:**
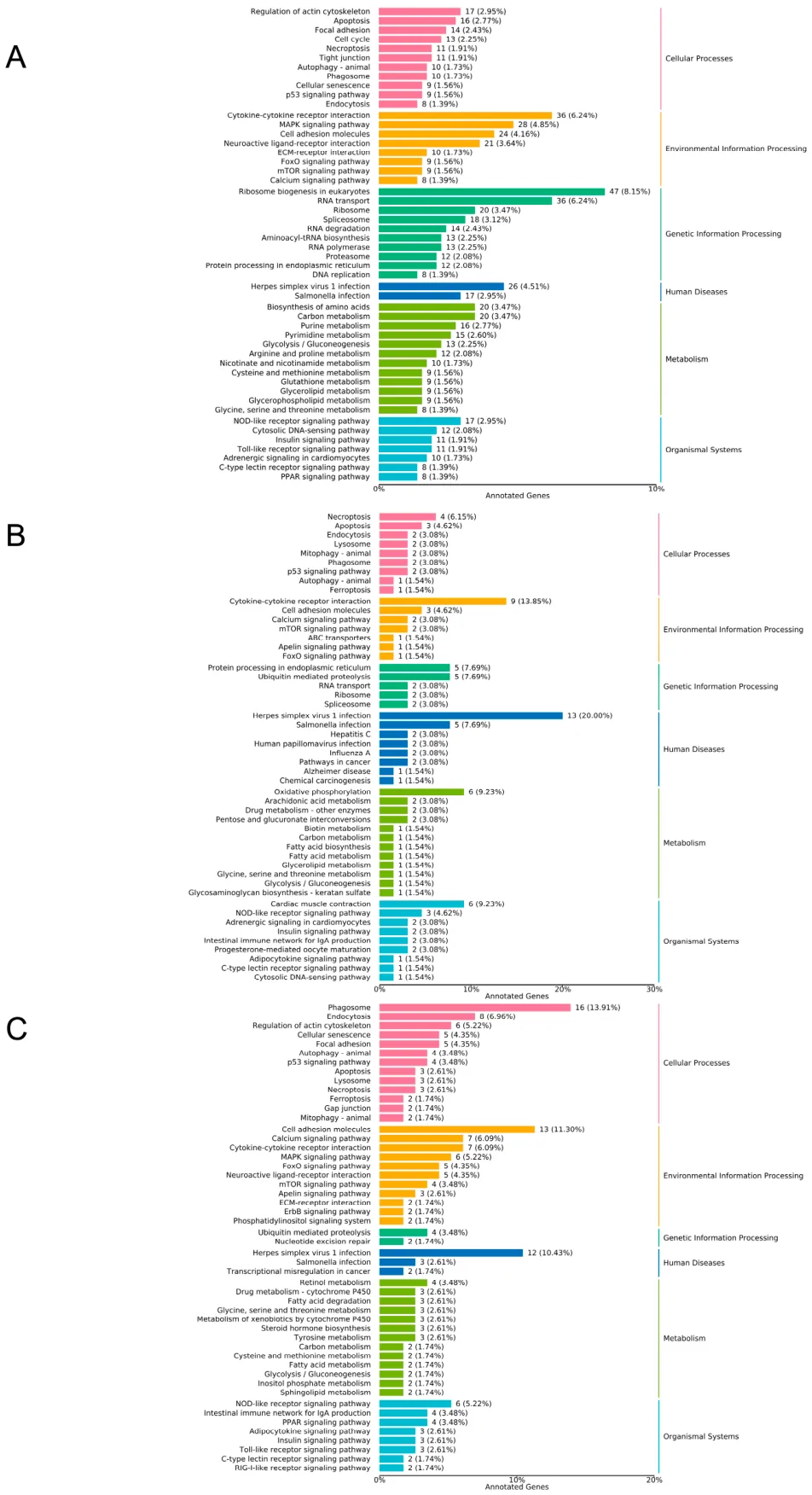
KEGG pathway enrichment of significantly upregulated genes in gills and intestine of vaccinated Asian seabass (*Lates calcarifer*) relative to control fish after bacterial challenge. (**A**) Gills after *F. covae* (Fc) immersion, (**B**) gills after *Vh/Vv/Pd* immersion, and (**C**) intestine after *Vh/Vv/Pd* injection. Pathway enrichment was performed using significantly upregulated transcripts meeting the predefined differential-expression criteria of FDR-adjusted *p* < 0.05 and absolute fold change > 1.5.

KEGG enrichment for upregulated gill genes after *Fc* immersion showed upregulation centered on genetic-information processing, led by ribosome biogenesis (8.15%) and RNA transport (6.24%), with supportive ribosome/spliceosome, proteasome and ER-processing, alongside moderate innate signaling (cytokine–cytokine receptor, NOD-like, TLR) and cytoskeleton/adhesion remodeling (Figure 3A). In gills after *Vh/Vv/Pd* immersion, immune–mitochondrial activation dominated, with cytokine–cytokine receptor interaction (13.85%) and oxidative phosphorylation (9.23%) most enriched; ER/proteasome pathways (7.69%) and vesicle–lysosome/cell-death programs accompanied modest RNA-handling signals (Figure 3B). The enrichment of upregulated genes in intestine after *Vh/Vv/Pd* injection indicated vesicular trafficking and receptor signaling prevailed, led by phagosome (13.91%) and cell adhesion molecules (11.30%), with endocytosis/cytokine–calcium signaling, MAPK/NOD hubs, and autophagy–lysosome/proteostasis modules indicating heightened endo-lysosomal turnover and tuned stress responses (Figure 3C).

### 2.5. Differentially Expressed Upregulated Genes

Differentially expressed genes (DEGs) were identified using DESeq2 with an FDR-adjusted *p* < 0.05 and an absolute fold change > 1.5. In gills following *F. covae* immersion challenge, 2399 DEGs were identified, including 1324 upregulated and 1075 downregulated genes. In gills following the mixed *Vh/Vv/Pd* immersion challenge, 274 DEGs were detected, comprising 155 upregulated and 119 downregulated genes. In intestine following the mixed *Vh/Vv/Pd* intraperitoneal challenge, 607 DEGs were identified, including 278 upregulated and 329 downregulated genes.

Across comparisons, three conditions were profiled: gills with *F.* covae immersion, gills after *Vh/Vv/Pd* immersion, and intestine after *Vh/Vv/Pd* injection. Vaccinated fish consistently showed greater counts of upregulated immune genes relative to controls in all groups, with the gill tissue under immersion challenge displaying the largest immune gene surge.

**Figure 4:**
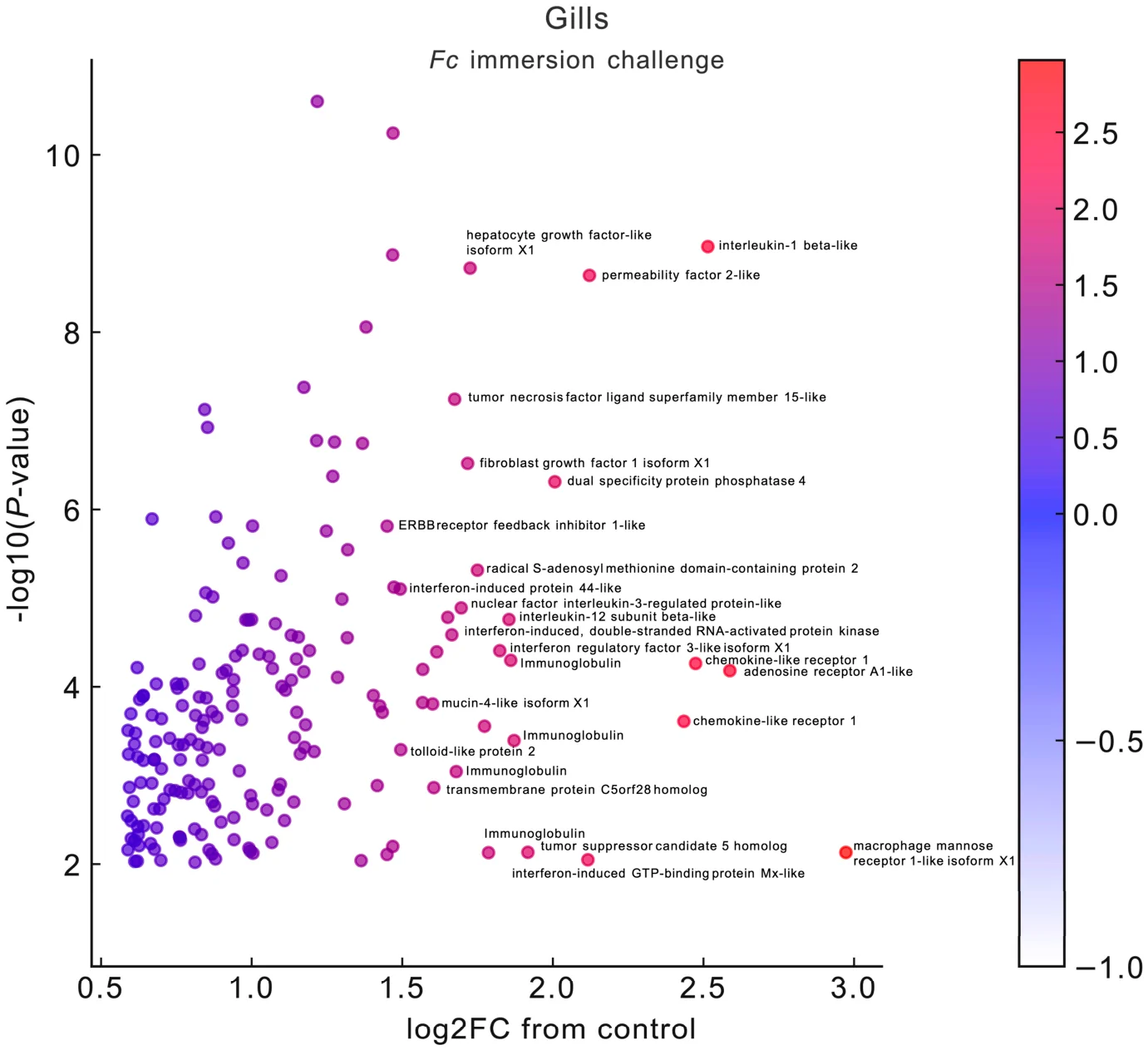
Volcano plots of differentially expressed immune-related transcripts in gills and intestine of vaccinated Asian seabass (*Lates calcarifer*) relative to control fish after bacterial challenge. Each panel shows log2 fold change (log2FC) versus −log10(*p*) for gills after *F. covae* (*Fc*) immersion. Each point represents a transcript, with significantly differentially expressed genes. Immune-related genes are highlighted, and key candidates are annotated by name. Differentially expressed genes (DEGs) were identified using DESeq2 with an FDR-adjusted *p* < 0.05 and an absolute fold change > 1.5.

In gills challenged by *F.* covae, vaccinated fish displayed a broad right-shift on the volcano plot with multiple highly significant immune and epithelial mediators upregulated (to about log2FC ~2.5 and −log10*P* ~10). Hallmark interferon-stimulated and signaling genes increased, including interferon-induced protein 44-like, Mx-like, PKR, and IRF3-like, together with pro-inflammatory and chemotactic cues (interleukin-1β-like, interleukin-12β-like, TNFSF15-like, chemokine-like receptor 1). Antigen handling and endocytic markers (macrophage mannose receptor 1-like, ERBB receptor feedback inhibitor 1-like, DUSP4) were elevated, as were barrier/ECM remodeling factors (mucin-4-like, tolloid-like protein 2, FGF1, HGF-like). Multiple immunoglobulin features also rose, representing activation of mucosal humoral responses (Figure 4).

**Figure 5:**
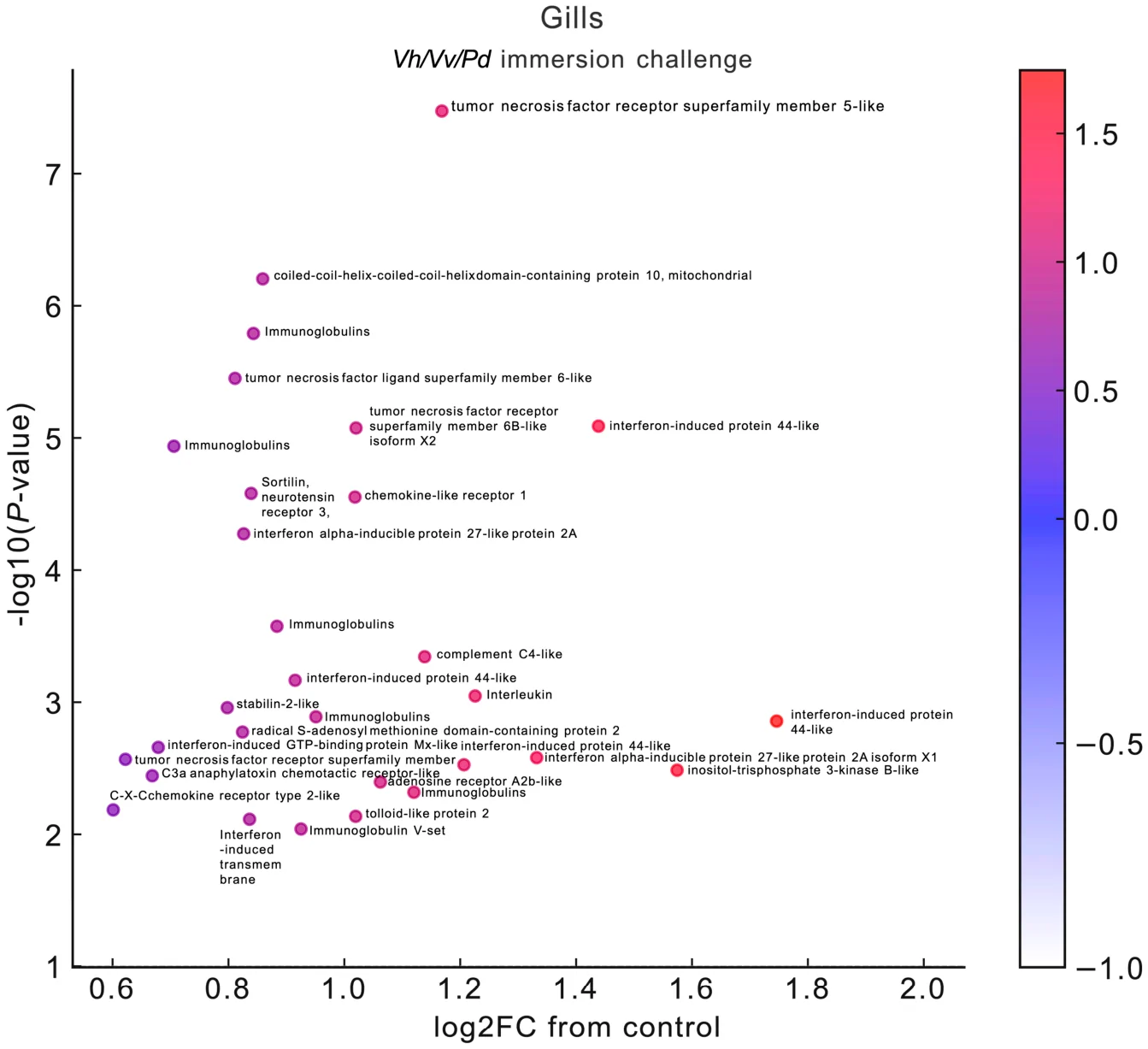
Volcano plots of differentially expressed immune-related transcripts in gills and intestine of vaccinated Asian seabass (*Lates calcarifer*) relative to control fish after bacterial challenge. Each panel shows log2 fold change (log2FC) versus −log10(*p*) for gills after *Vh/Vv/Pd* immersion. Each point represents a transcript, with significantly differentially expressed genes. Immune-related genes are highlighted, and key candidates are annotated by name. Differentially expressed genes (DEGs) were identified using DESeq2 with an FDR-adjusted *p* < 0.05 and an absolute fold change > 1.5.

In gills under *Vh/Vv/Pd* immersion, vaccination produced a clear right-shift on the volcano plot with many features surpassing significance (up to ~7 on −log10(*p*)) and moderate effect sizes (log2FC ~0.8–2.0). Prominent interferon-stimulated genes increased, including interferon-induced protein 44-like (IFI44-like), interferon alpha-inducible protein 27-like (IFI27-like), interferon-induced transmembrane (IFITM) family members, and Mx-like, alongside chemotactic and inflammatory signals (C-X-C chemokine receptor type 2-like, chemokine-like receptor 1, IL-1-related, TNFRSF5/6B. Complement and danger-sensing pathways were elevated (C3a receptor-like, complement C4-like), together with epithelial remodeling factors (tolloid-like protein 2, stabilin-2-like, sortilin/neurotensin receptor 3. Multiple immunoglobulin features were among the most increased, consistent with boosted mucosal humoral activity. Collectively, these data indicate coordinated induction of IFN-driven innate readiness, leukocyte recruitment, complement activation, and antibody responses in vaccinated gills (Figure 5).

**Figure 6:**
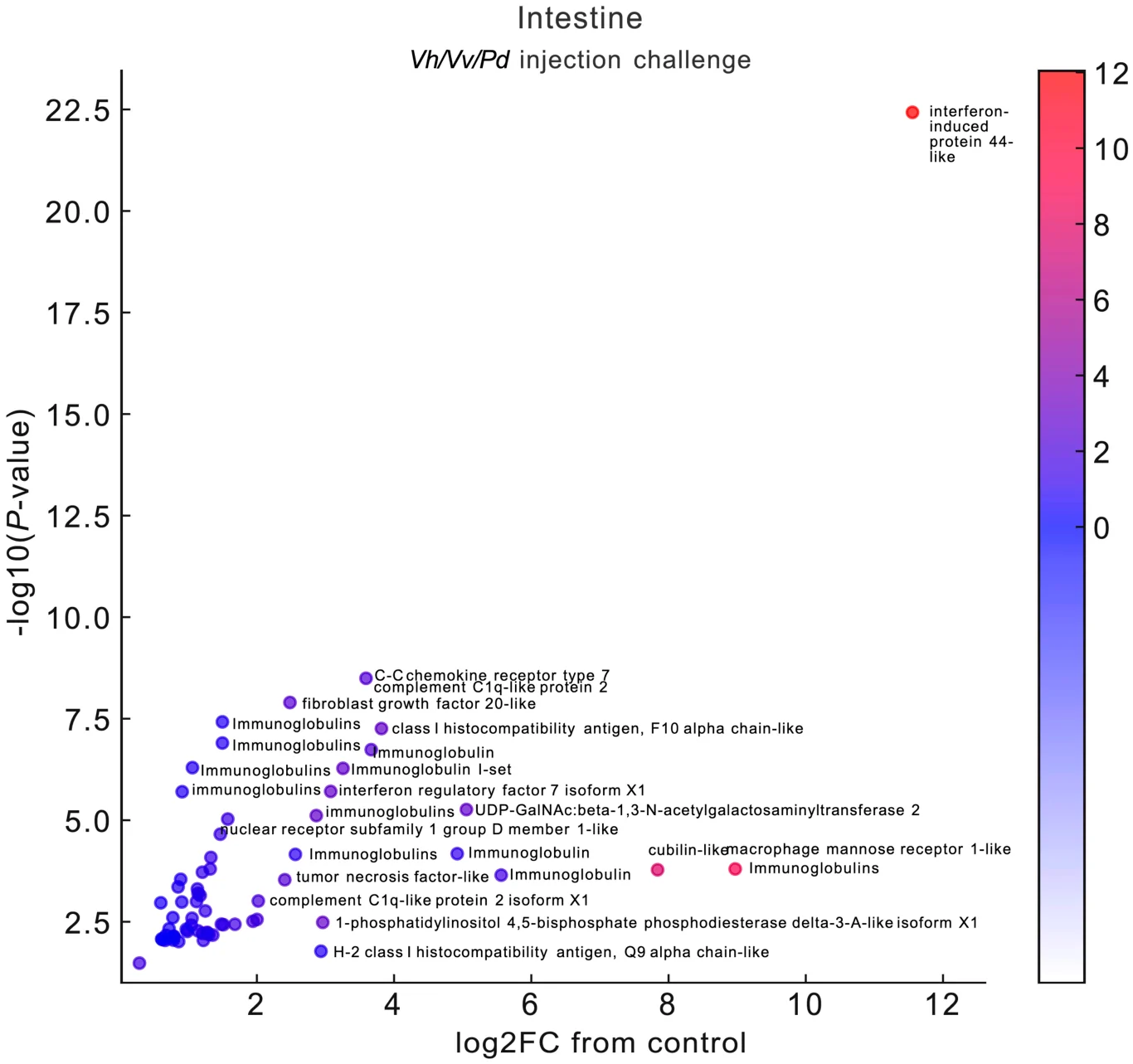
Volcano plots of differentially expressed immune-related transcripts in gills and intestine of vaccinated Asian seabass (*Lates calcarifer*) relative to control fish after bacterial challenge. Each panel shows log2 fold change (log2FC) versus −log10(*p*) for intestine after *Vh/Vv/Pd* injection. Each point represents a transcript, with significantly differentially expressed genes. Immune-related genes are highlighted, and key candidates are annotated by name. Differentially expressed genes (DEGs) were identified using DESeq2 with an FDR-adjusted *p* < 0.05 and an absolute fold change > 1.5.

In intestine under *Vh/Vv/Pd* injection, vaccination produced a strong right-shift on the volcano plot with many features achieving high significance (up to ~20 on −log10(*p*)) and large positive effects (log2FC up to ~12 for immunoglobulin features). Recurrently elevated markers included multiple immunoglobulin chains and I-set domains, complement C1q-like proteins, interferon axis genes (IFI44-like, IRF7 isoform X1), antigen-presentation signatures (class I histocompatibility chains), leukocyte-trafficking cues (CCR7), and endocytic/epithelial modulators (macrophage mannose receptor 1-like, cubilin-like, tolloid-adjacent remodeling factors). There were additional increases in FGF20-like, UDP-GalNAc transferase 2, and PLC-δ3-A-like (Figure 6).

### 2.6. Identification of Top Upregulated Immune-Related Genes

**Figure 7:**
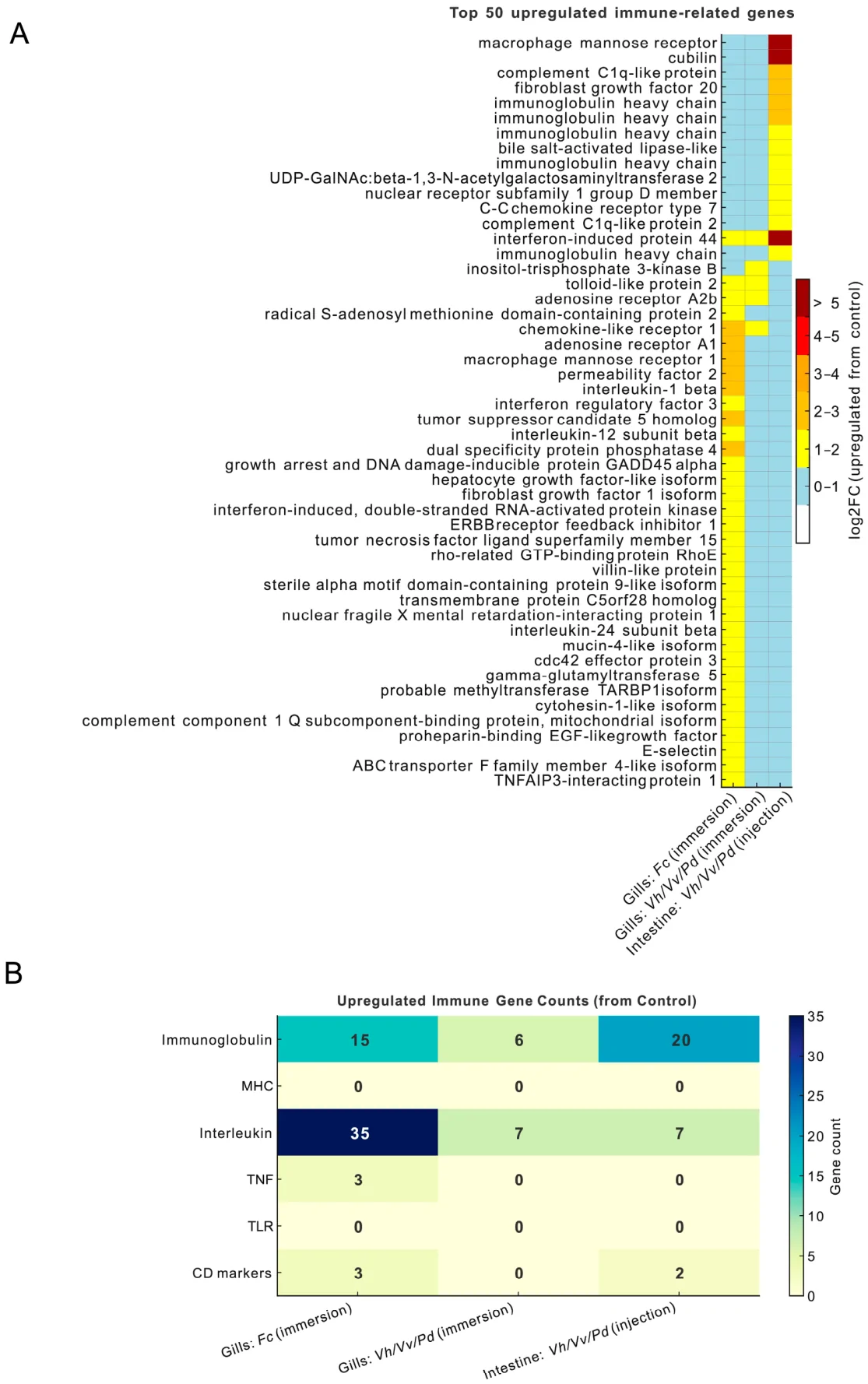
Summary of top upregulated immune-related genes in mucosal tissues of vaccinated Asian seabass (*Lates calcarifer*) following bacterial challenge. (**A**) Heatmap of the top 50 significantly upregulated immune-related transcripts in gills after *F. covae* (Fc) immersion, gills after *Vh/Vv/Pd* immersion, and intestine after *Vh/Vv/Pd* injection, shown as log2 fold change (log2FC) values relative to the corresponding unvaccinated controls. (**B**) The number of significantly upregulated immune-related transcripts assigned to major functional categories for each tissue/challenge group. Differentially expressed genes (DEGs) were identified using DESeq2 with an FDR-adjusted *p* < 0.05 and an absolute fold change > 1.5.

The top 50 immune-related DEGs showed strong mucosal humoral and pattern-recognition activation. In gills after *Fc* immersion, immunoglobulin heavy chains and complement components were prominently upregulated together with macrophage mannose receptor and Toll-like proteins, indicating enhanced antigen capture and classical complement signaling (Figure 7A).

Gills after *Vh/Vv/Pd* immersion exhibited broader innate immune-associated signaling, with increased cytokine and chemokine receptors, interferon-inducible genes, and RNA-activated kinase, alongside adhesion molecules and protease regulators. These changes are consistent with molecular pathways involved in chemokine signaling, leukocyte trafficking, and antimicrobial responses, but leukocyte recruitment was not measured directly (Figure 7A).

In intestine after *Vh/Vv/Pd* injection, phagocytic receptors and interferon-stimulated genes were strongly induced together with mucin- and epithelial-junction-associated markers. These changes are consistent with molecular processes related to phagosome activity, epithelial organization, and inflammatory regulation, but functional barrier integrity was not directly assessed (Figure 7A).

Vaccination preferentially boosted immunoglobulin and interleukin transcripts among the top 50 immune genes, with strong tissue- and challenge-specific patterns. In gills challenged by *F. covae* immersion, immunoglobulin features were prominent (*n* = 15) and interleukins were the most numerous (*n* = 35), accompanied by smaller TNF signals *(n* = 3) and low-level CD markers (*n* = 3). Under *Vh/Vv/Pd* immersion in gills, immunoglobulins (*n* = 6) and interleukins (*n* = 7) remained increased, while TNF, TLR, and MHC categories were not represented. Intestine under *Vh/Vv/Pd* injection showed the strongest humoral skew with immunoglobulins dominating (*n* = 20) and modest interleukin (*n* = 7) and CD marker *(n* = 2) increases, again without detectable TLR or MHC entries among the top 50 (Figure 7B).

### 2.7. Proteomic Analysis

Proteomic analysis provided complementary evidence for several molecular patterns identified in the transcriptomic dataset. In particular, vaccinated fish showed a higher abundance of immunoglobulin chains, complement components, lysosomal cathepsins, heat shock and redox chaperones, together with changes in ribosomal, cytoskeletal, and adhesion-associated proteins. These protein-level responses were broadly consistent with functional categories identified by transcriptomic analysis, although direct correspondence between individual transcript and protein abundance was not assumed.

**Figure 8:**
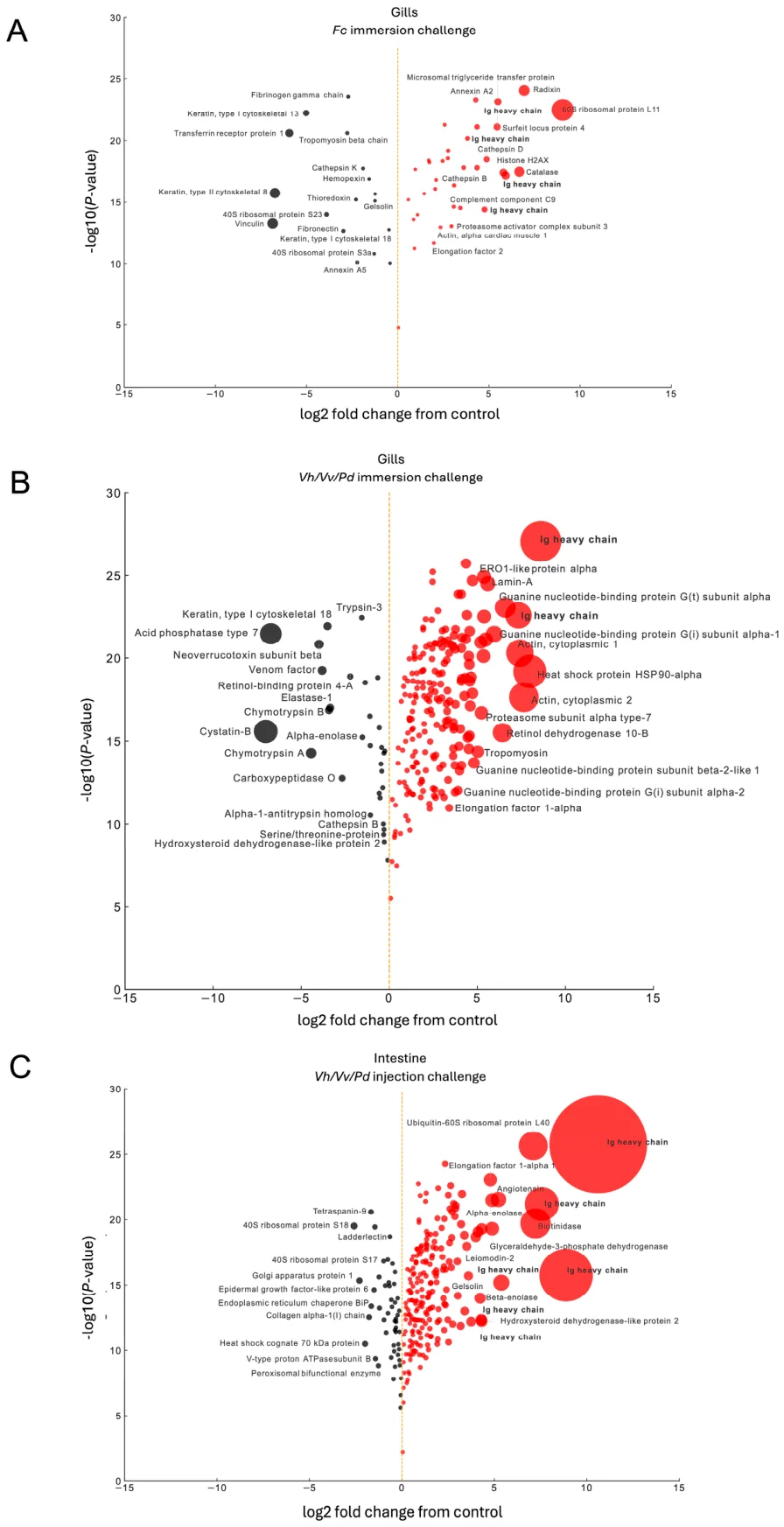
Volcano plots of differentially abundant proteins in mucosal tissues of vaccinated Asian seabass (*Lates calcarifer*) relative to control fish following bacterial challenge. Panels show log2 fold change in vaccinated versus control fish on the x axis and −log10(*p* value) on the y axis for (**A**) gills after *F. covae* (*Fc*) immersion, (**B**) gills after *Vh/Vv/Pd* immersion, and (**C**) intestine after *Vh/Vv/Pd* injection. Proteins with upregulated expression in vaccinated fish are located on the right side of each plot (red color), whereas those with downregulated expression are on the left (black color).

In the proteome comparison of gills after *Fc* immersion, the volcano plot shows a broad right-shift with many proteins passing significance (up to ~30 on −log10*P*) and large positive effects (log2FC frequently > 3). The volcano plot showed a pronounced right shift, with many proteins exceeding stringent significance thresholds. Immunoglobulin heavy-chain were markedly increased, together with core translational components (60S L11, 40S S3, 40S SA, 40S S18), cytoskeletal–membrane organizers (radixin, annexin A2), oxidative-stress defense (catalase), lysosomal protease activity (cathepsin D), chromatin/DNA-damage response (histone H2AX), and secretory trafficking (SURF4). In contrast, only a few proteins were strongly decreased, led by vinculin, keratin-8, and the transferrin receptor. Collectively, these changes indicated that vaccination enhanced mucosal humoral immunity, protein synthesis, membrane–actin remodeling, antioxidant and lysosomal capacity, and secretory readiness, while reducing selected adhesion and iron-uptake modules consistent with a reinforced effector-ready state (Figure 8A and Table S3).

In the gills after *Vh/Vv/Pd* immersion, the proteome showed a clear right-shift on the volcano plot, with many features surpassing stringent significance. Upregulated proteins predominantly reflected immune–mitochondrial activation and trafficking: cytokine and chemokine receptors and interferon-responsive factors were increased together with immunoglobulin heavy-chain species, complement components, and phagosome–lysosome machinery; oxidative phosphorylation and ER/proteasome modules were also elevated, indicating boosted respiration, protein processing, and turnover. Actin–adhesion remodeling and endocytosis/mitophagy signals accompanied these changes, consistent with intensified pathogen recognition and clearance. In contrast, the downregulated set was small and centered on select adhesion or membrane-transport elements, suggesting a shift away from structural maintenance toward an effector-ready mucosal state (Figure 8B and Table S4).

In the intestine after *Vh/Vv/Pd* injection, the proteomic volcano plot showed a clear right-shift with many proteins surpassing stringent significance and exhibiting large positive effects (several with log2FC > 3 and −log10*P* approaching 30). Upregulated features encompassed immunoglobulin heavy-chain species and complement components, interferon-responsive factors, cytokine/chemokine receptors, and phagosome–lysosome machinery, together with oxidative-phosphorylation subunits, ER chaperones/proteasome subunits, and endocytosis/actin-adhesion remodelers—consistent with intensified recognition, processing, and clearance of pathogens. Downregulated proteins were few and modest, largely confined to selected adhesion or membrane-transport factors, indicating a shift from structural maintenance toward an effector-ready mucosal state (Figure 8C and Table S5).

Because transcript abundance does not necessarily predict corresponding protein abundance, the transcriptomic and proteomic datasets were interpreted as complementary molecular layers rather than as directly equivalent measurements. Agreement in selected immune and cellular functional categories therefore provides supporting evidence for these responses, whereas differences between the two datasets may reflect post-transcriptional regulation, translation efficiency, protein stability and turnover, or differences in analytical sensitivity.

### 2.8. Pathway Signatures of Immune Genes in Vaccinated Asian Seabass Following Bacterial Challenge

#### 2.8.1. Cell Adhesion Molecules

**Figure 9:**
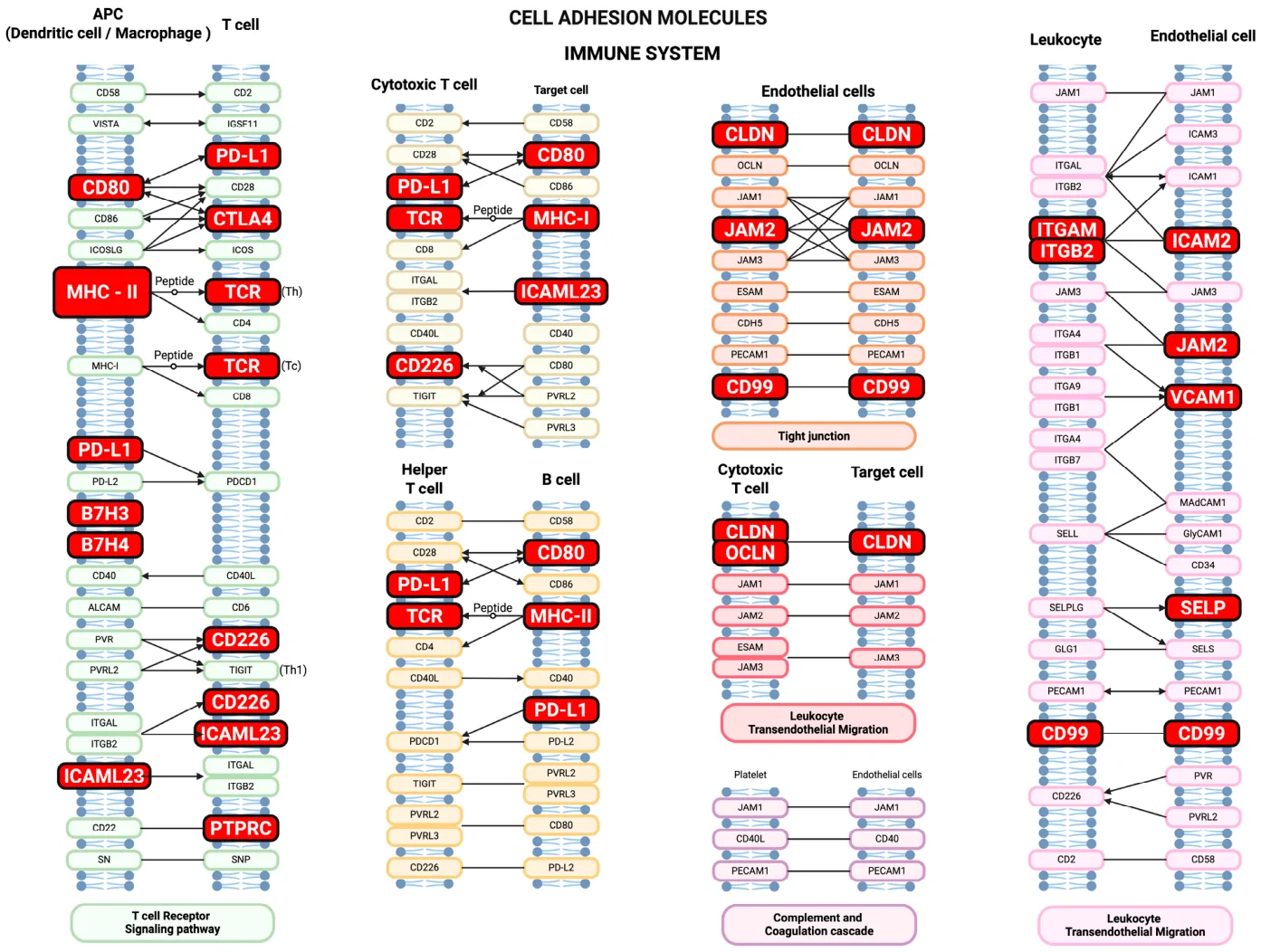
Integrated model of endothelial adhesion, leukocyte trafficking, and T cell activation pathways engaged in vaccinated Asian seabass after bacterial challenge. Schematic representation of the major cell adhesion and immune signaling modules inferred from significantly upregulated genes and proteins in gills after *F. covae* (*Fc*) and *Vh/Vv/Pd* immersion.

Vaccinated fish mounted strong immune responses across all bacterial challenge models, including immersion challenge with *F. covae* and challenges with *Vibrio* spp. and *P. damselae*. The consistent upregulation of target genes (*p* < 0.05) indicated broad activation of protective mechanisms against bacterial infection. Pathway mapping identified a set of adhesion- and lymphocyte-associated molecules consistent with molecular processes involved in leukocyte trafficking and immune-cell interaction. The detected features included MHC-I/II- and TCR-associated components, CD80/CD28 and CD40/CD40L costimulatory molecules, as well as ICAM-like molecules, VCAM1, ITGAL, ITGAM, ITGB2, JAM2, CD99, and SELP. Together, these changes indicate enrichment of molecular pathways associated with leukocyte adhesion, transendothelial migration, and lymphocyte signaling in vaccinated fish. Because leukocyte migration and T cell activity were not measured directly, these findings are interpreted as molecular signatures consistent with these processes rather than direct evidence of enhanced cellular trafficking or T cell activation (Figure 9).

#### 2.8.2. Intestinal Immune Network for Igs Production

**Figure 10:**
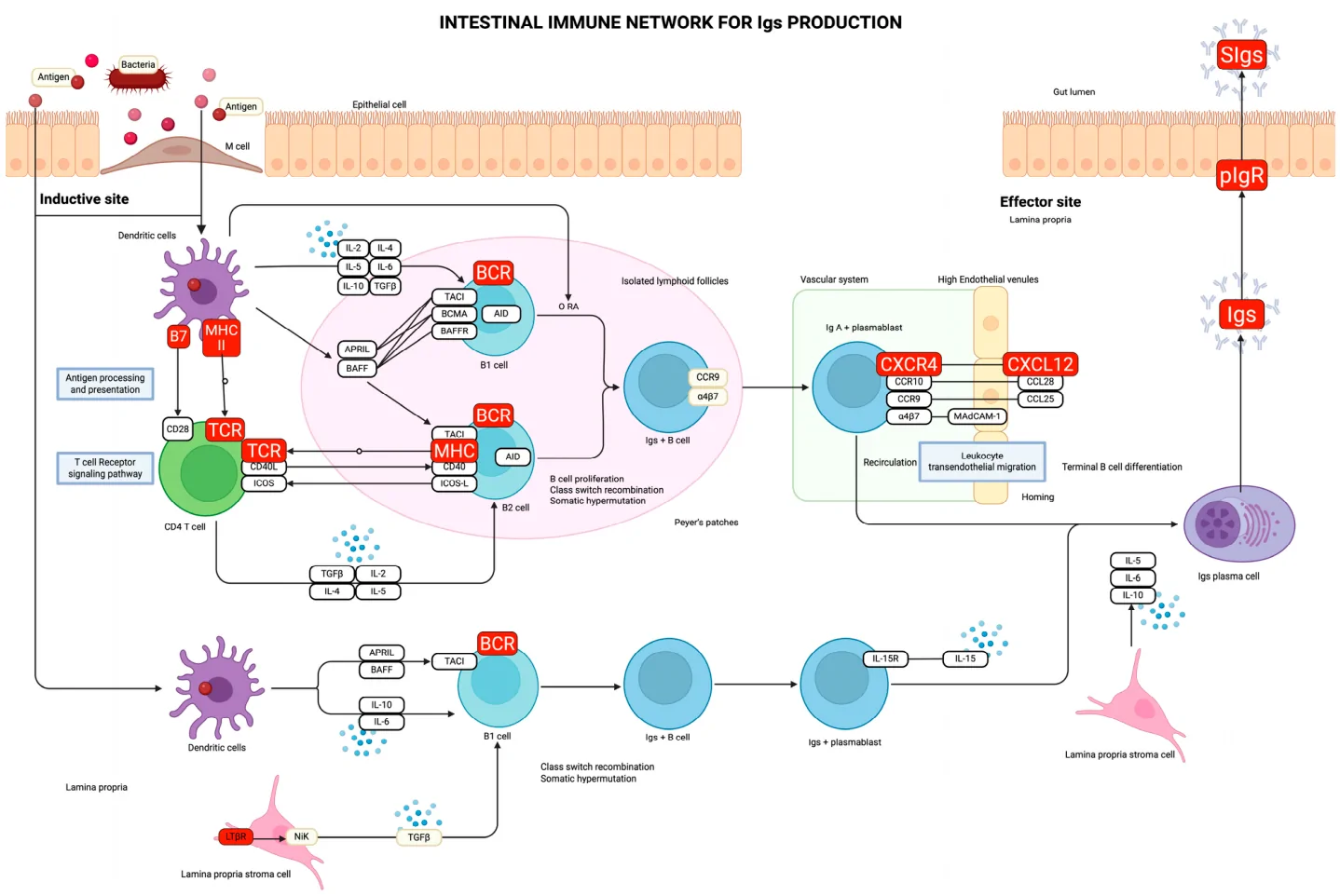
Integrated intestinal immune network for immunoglobulin production in vaccinated Asian seabass (*Lates calcarifer*). Schematic representation of the KEGG “intestinal immune network for Igs production” pathway, adapted to illustrate key cellular interactions and signaling modules inferred from differentially expressed genes and proteins in intestine after *Vh/Vv/Pd* injection.

In intestine following the mixed *Vh/Vv/Pd* intraperitoneal challenge, the enriched immune network contained multiple molecules associated with humoral immune organization. These included MHC-II/TCR-associated components, CD28-related costimulation, BCR-associated molecules, the CXCR4-CXCL12 chemokine axis, multiple immunoglobulin transcripts, and pIgR-associated epithelial transport. Collectively, these molecular features are consistent with pathways involved in B-cell signaling, lymphocyte homing, immunoglobulin production, and epithelial transport of polymeric immunoglobulins. However, because antigen-specific antibody secretion, B-cell differentiation, and pIgR-mediated transcytosis were not directly quantified, these findings are interpreted as pathway-level molecular signatures rather than direct functional demonstration of these processes (Figure 10).

### 2.9. RNA-Seq Validation of Expression by qRT-PCR

**Figure 11:**
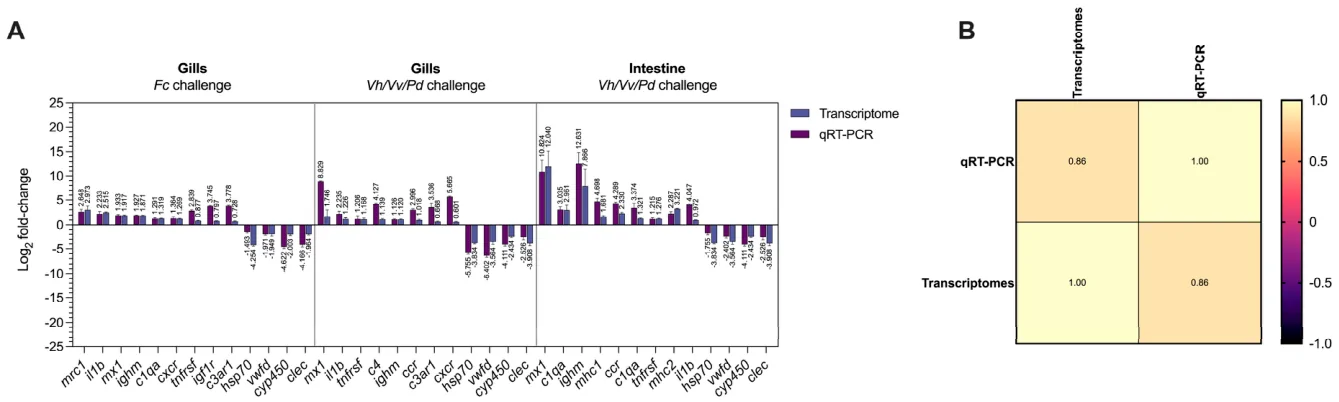
Validation of RNA-seq-based differential expression by qRT-PCR in mucosal tissues of vaccinated Asian seabass (*Lates calcarifer*). (**A**) Comparison of log2 fold change in vaccinated group with control across three experimental groups: gills after *F. covae* (*Fc*) immersion, gills after *Vh/Vv/Pd* immersion, and intestine after *Vh/Vv/Pd* injection. (**B**) Correlation between log2 fold change values determined by RNA-seq and qRT-PCR for all genes and tissue/challenge combinations, illustrating strong concordance between the two methods. Data are shown as mean ± SD of biological replicates (*n* = 3).

qRT-PCR analysis corroborated the RNA-seq calls across all tissues and challenges. In gills after *Fc* immersion, key targets such as *mrc1*, *il1b*, *mx1*, *c1qbp*, *cxcr4*, *tnfrsf11b*, *igf1r*, and *c3ar1* showed concordant upregulation with comparable effect sizes. In gills after *Vh/Vv/Pd* immersion, interferon- and cytokine-axis genes (*ifi44*, *ifi27l2a*, *il*, *tnfrsf5*, *c4*, *tnfrsf6b*, *cmklr1*, *c3ar1*, *cxcr2*) validated the RNA-seq trend. In intestine after *Vh/Vv/Pd* injection, an interferon–complement/antigen-presentation signature was confirmed, including *ifi44*, *c1ql2*, *ighv2*, *mhc-i* (*h2-q9*), *ccr7*, *irf7*, and *il21r*. Across datasets, cross-platform agreement was strong (RNA-seq *vs.* qRT-PCR correlation *r* = 0.86; within-platform replicates *r* = 1.00). Together, the validation supports the robustness of the RNA-seq findings and the vaccine-driven activation of mucosal immune pathways (Figure 11).

### 2.10. Effect of Sequential Vaccination over Five Months on Survival Following Bacterial Challenge

**Figure 12:**
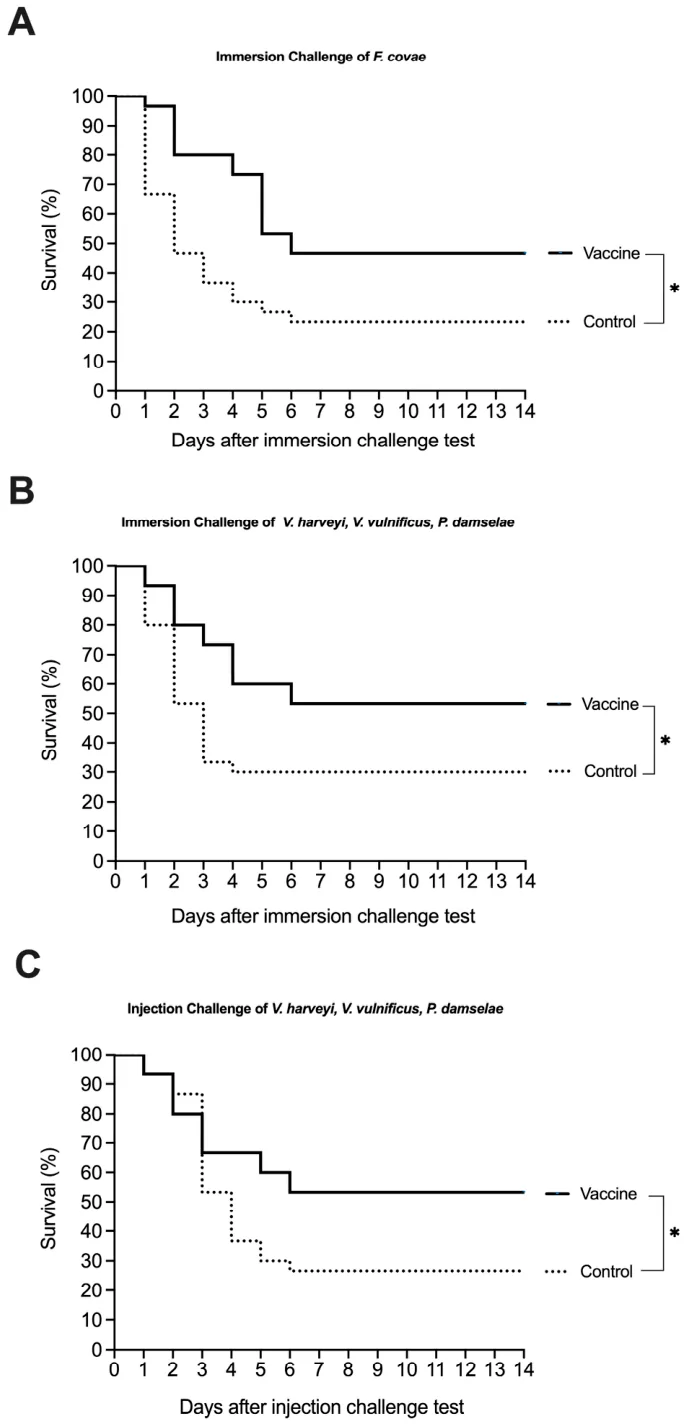
Kaplan–Meier survival analysis of Asian seabass following bacterial challenge after sequential vaccination. Cumulative survival of control and vaccinated fish was monitored over 14 days after (**A**) *F. covae* immersion challenge, with final survival 23.33% and 46.67%, respectively; (**B**) mixed *V. harveyi/V. vulnificus/P. damselae* immersion challenge, with final survival 30.00% and 53.33%, respectively; and (**C**) mixed *V. harveyi/V. vulnificus/P. damselae* intraperitoneal challenge, with final survival 26.67% and 53.33%, respectively. Survival curves were compared using the log-rank (Mantel–Cox) test (*n* = 30/group; three replicate tanks with 10 fish per tank). Asterisks (*) indicate significant differences between groups (*p* < 0.05).

Sequential nano-immersion priming followed by oral booster vaccination significantly improved the survival of Asian seabass across both immersion and injection challenge models. Following *F. covae* immersion challenge, the vaccinated group showed a significantly higher final cumulative survival of 46.67% compared with 23.33% in the control group, corresponding to an RPS of 30.43% (*p* < 0.05; Figure 12A). Similarly, following the mixed immersion challenge with *V. harveyi*, *V. vulnificus*, and *P. damselae*, final cumulative survival was significantly higher in the vaccinated group (53.33%) than in the control group (30.00%), with an RPS of 33.33% (*p* < 0.05; Figure 12B). In the mixed intraperitoneal challenge, vaccinated fish also showed significantly higher final cumulative survival (53.33%) than control fish (26.67%), corresponding to an RPS of 36.36% (*p* < 0.05; Figure 12C).

## 3. Discussion

This study used integrated transcriptomic and proteomic profiling to dissect how a long-term sequential mucosal vaccination regimen reshapes gill and intestinal immunity in Asian seabass challenged with four major bacterial pathogens. By combining nanoemulsion immersion priming with multivalent oral hydrogel boosting, we show that vaccinated fish mount a coordinated response across mucosal tissues that couples translational activation, lysosome and phagosome remodeling, interferon and cytokine signaling, endothelial adhesion, and robust humoral deployment. The consistency between RNA-seq, proteomics, and qRT-PCR verification indicates that the observed signatures reflect true vaccine-driven remodeling of mucosal immune networks.

PCA clearly separated vaccinated from control fish in both gill and intestinal tissues for all challenge models, highlighting a strong and coherent vaccination effect [19,22,44]. This extends earlier work on nanoemulsion immersion and oral hydrogel vaccines in Asian seabass, which reported enhanced antibody titers and protection but were limited to selected markers. The present data show that noninvasive prime–boost schedules do not simply increase a few cytokines or antibodies, but globally reprogram mucosal gene expression and protein abundance [28,45].

Despite shared vaccination history, the three tissue/challenge combinations retained distinct expression patterns, consistent with anatomical specialization of mucosa-associated lymphoid tissues in teleosts [12,23,46]. Gills, as a primary portal for waterborne pathogens and environmental stressors, showed particularly strong enrichment of interferon-stimulated genes, chemokine receptors, and epithelial remodeling factors after both *F. covae* and *Vibrio/Photobacterium* immersion [21,22]. Intestine challenged by injection was dominated by massive upregulation of immunoglobulin chains and complement components, in line with its role as a central site for antibody production and oral vaccine responsiveness [28,31].

Across tissues, upregulated genes converged on ribosome biogenesis, RNA processing, and translational machinery, accompanied by lysosome, phagosome, and proteasome pathways. This combination suggests that vaccinated mucosae enter a metabolically active, protein-synthesis-competent state that supports continuous production of receptors, signaling mediators, and effector molecules rather than a short-lived burst of activation [28,30,47]. Elevated lysosomal and endosomal terms, together with cathepsin and phagosome enrichment, indicate increased capacity for antigen uptake, processing, and pathogen degradation [48,49,50].

Similar transcriptomic patterns have been observed in teleost gut and spleen following bacterial infection or probiotic stimulation, where ribosome and lysosome pathways co-enrich with immune signaling [48,51,52,53]. In our dataset, these processes were coupled to cytokine receptor interaction, NOD-like and Toll-like receptor signaling, and oxidative phosphorylation pathways, consistent with an energy-intensive, receptor-driven response that supports sustained antimicrobial defense without collapsing core metabolism [30,50,51].

A common signature across tissues was the robust induction of interferon-stimulated genes (IFI44-like, IFI27-like, IFITM family, MX), pattern-recognition molecules (macrophage mannose receptor 1-like, chemokine receptors), complement components (C1q-, C3-, C4-like), and epithelial mediators (mucin-4-like, tolloid-like proteases, stabilin-2, sortilin). Interferon programs are increasingly recognized as central to antiviral and antibacterial defense in fish mucosa, regulating both innate effectors and adaptive activation [54,55,56]. The concomitant enhancement of complement and chemokine pathways suggests that vaccinated seabass are primed for rapid detection, opsonization, and recruitment of leukocytes to infected sites [48,57].

At the epithelial level, increased expression of mucins, junction-associated molecules, and extracellular-matrix regulators indicates transcriptional remodeling of barrier-related pathways. Previous reviews of teleost mucosal immunity emphasize that gill and gut epithelia integrate structural, innate, and adaptive mechanisms to balance microbial exclusion with tolerance to commensals [31,58]. However, because epithelial permeability, junctional integrity, and histological protection were not measured directly, the present data should be interpreted as molecular signatures associated with epithelial responses rather than evidence of improved barrier function [25,57].

Intestinal samples after *Vibrio/Photobacterium* injection displayed the most striking humoral skew, with multiple immunoglobulin heavy chains and I-set domain-containing genes showing very high log2 fold changes, supported by C1q-like complement, CD markers, and cytokine receptors. This echoes KEGG “intestinal immune network for IgA production” architecture, where B cell activation, class switching, homing, and epithelial transcytosis collaborate to deliver large quantities of non-inflammatory antibodies to the lumen. Although teleosts lack IgA, IgM and IgT/IgZ fulfill analogous functions at mucosal surfaces [25,46,59].

The integrated network in our study highlighted MHC-II–TCR engagement, BCR signaling, BAFF/APRIL axes, chemokine-guided homing, and pIgR-mediated export, strongly indicating that the sequential vaccination regimen effectively drives T–B collaboration and secretory antibody production [20,60,61]. This is consistent with oral hydrogel vaccine work in seabass, where multivalent *Vibrio* antigens enhanced intestinal Ig expression and protected fish against vibriosis [19,62]. By including *F. covae* antigens and extending the schedule to five months, the present protocol appears to generalize this mucosal antibody network across freshwater and marine pathogens [25,41,63].

Label-free proteomics provided independent evidence that transcriptional changes translated into effector deployment. Vaccinated gills and intestine showed an increased abundance of immunoglobulin heavy chains, complement proteins, cathepsins, and oxidative-stress chaperones such as HSP70 and HSP90, together with ribosomal proteins, actin-associated organizers, and adhesion molecules [25,57,62].

The transcriptomic and proteomic datasets provided complementary perspectives on the mucosal response to the sequential vaccination regimen. Selected functional patterns were represented at both molecular levels, including immunoglobulin-related responses, complement components, lysosomal proteins, stress-response factors, and cytoskeletal or adhesion-associated molecules. These similarities strengthen the interpretation of these biological processes, but they should not be considered evidence of complete transcript–protein concordance. Differences between transcript and protein abundance can arise from post-transcriptional regulation, translation efficiency, protein stability and turnover, and temporal differences between transcription and protein accumulation. Therefore, the proteomic findings are interpreted as complementary support for selected transcriptomic patterns rather than as quantitative validation of the entire transcriptomic response [23,39,46,48,64].

Downregulation of a relatively small set of structural and transport proteins (for example, vinculin, selected keratins, transferrin receptor) may reflect a reallocation of resources from growth and baseline adhesion toward immune activity and controlled remodeling [19]. Importantly, there was no broad suppression of mitochondrial or core metabolic proteins, suggesting that vaccinated fish maintain metabolic resilience while elevating immune functions [20,63].

Pathway integration emphasized endothelial adhesion molecules, integrins, selectins, and platelet–endothelial contacts as a central axis connecting transcriptional and proteomic data. In mammals and domestic animals, leukocyte–endothelial interactions are orchestrated by selectins, ICAMs, VCAM1, JAMs, CD99, and integrins such as LFA-1 and Mac-1, and are essential for extravasation and trafficking [65,66]. Our data suggest that analogous modules operate in seabass mucosa, linking vascular activation to T cell costimulation and B cell help [9,67,68].

Upregulation of ICAM/VCAM, integrin alpha/beta pairs, MAdCAM1 and GlyCAM1, together with CD40–CD40L, CD80/CD86–CD28, ICOS–ICOSL, and PD-1/PD-L networks, indicates that vaccinated fish can more efficiently recruit leukocytes, form immune synapses, and fine-tune effector and regulatory T cell functions [69,70,71,72,73,74]. The convergence of endothelial adhesion, chemokine gradients, and immunoglobulin production supports a model in which the vaccine enhances both the delivery of immune cells to mucosal sites and their local activation and effector functions [6,23,75].

The four pathogens targeted in this work are major constraints in seabass and broader aquaculture. *Flavobacterium covae* is a key columnaris-causing species with growing recognition in freshwater systems [41,43,76], while *V. harveyi*, *V. vulnificus*, and *P. damselae* are well-known drivers of vibriosis and septicemia in brackish and marine production [40,42,77]. Developing a single platform capable of protecting across this ecological and taxonomic breadth is highly desirable [78].

By demonstrating that the same sequential regimen elicits strong yet context-specific responses in gills under freshwater-like and brackish challenges, and in intestine following systemic exposure, our data support the feasibility of broad-spectrum mucosal vaccines [25,28,44,45]. The shared core signatures (IFN, complement, immunoglobulin, adhesion) provide mechanistic candidates for correlates of protection that can be monitored in future efficacy and field trials.

The transcriptomic and proteomic datasets provided complementary but not fully overlapping views of the vaccination-associated response. Selected immune and cellular features, including immunoglobulin-related molecules, complement components, lysosomal proteins, stress-response factors, and cytoskeletal or adhesion-associated molecules, were represented at both molecular levels. However, transcript abundance does not necessarily translate proportionally into protein abundance because of post-transcriptional regulation, differences in translation efficiency, protein stability and turnover, and temporal differences between transcription and protein accumulation. Therefore, agreement between the two datasets should be interpreted as partial molecular concordance rather than direct validation of all transcriptomic changes.

A further limitation is that Western blot or other targeted protein-validation approaches were not performed, and formal quantitative transcript–protein integration was beyond the scope of the present study. In addition, gill and intestinal tissues were evaluated under different challenge routes, limiting direct tissue comparison, and intraperitoneal challenge does not fully reflect natural intestinal infection. Although the transcriptomic and proteomic datasets identified molecular signatures associated with leukocyte trafficking, lymphocyte signaling, immunoglobulin-related responses, and epithelial remodeling, these functional processes were not directly measured. In addition, antigen-specific antibody titers, bacterial load, histological protection, and functional leukocyte responses were not evaluated. Therefore, the transcriptomic and proteomic datasets should be interpreted as complementary molecular layers, and the proposed immune mechanisms should be regarded as biologically plausible interpretations of the observed molecular patterns rather than as direct validation of one another or direct evidence of functional immune activation.

## 4. Materials and Methods

### 4.1. Preparation of Bacterial Pathogens

Four bacterial pathogens associated with disease outbreaks in Asian seabass were included in this study: *Flavobacterium covae* (*Fc*), *Vibrio harveyi* (*Vh*), *Vibrio vulnificus* (*Vv*), and *Photobacterium damselae* (*Pd*). All strains were obtained from the bacterial culture collection of the Center of Excellence in Aquatic Animal Health Management (CE AAHM), Faculty of Fisheries, Kasetsart University, Bangkok, Thailand, and had originally been isolated from diseased Asian seabass. The strains used were *F. covae* AAHM-FCSB2401, *V. harveyi* AAHM-VHSB2402, *V. vulnificus* AAHM-VVSB2420, and *P. damselae* AAHM-PDSB2521. Bacterial identities were confirmed using *16S rRNA* gene sequencing.

For bacterial propagation, *F. covae* was grown in Shieh medium at 27 to 30 °C, whereas *V. harveyi*, *V. vulnificus*, and *P. damselae* were cultured in tryptic soy broth (TSB) supplemented with 2.0% NaCl at 30 to 32 °C for 24 h. Following incubation, the bacterial cells were collected by centrifugation at 5000× *g* for 15 min and rinsed twice with sterile 0.85% NaCl solution, pH 7.4. The harvested bacteria were then inactivated with 1% formaldehyde at 4 °C for 48 h. After inactivation, the bacterial suspensions were washed three times to eliminate residual formaldehyde and resuspended in sterile saline prior to vaccine preparation. The bacterial concentration was adjusted to 1 × 10^9^ CFU/mL based on optical density measurement at 600 nm using a spectrophotometer (Thermo Fisher Scientific, Waltham, MA, USA), and the standardized suspensions were kept for subsequent vaccine formulation.

### 4.2. Vaccine Formulation of Bivalent Nanoemulsion (Immersion) and Multivalent Hydrogel (Oral) Vaccines

A portion of each formalin-inactivated bacterial suspension was subjected to sonication at 40% amplitude for 15 min to improve bacterial cell dispersion and facilitate antigen exposure. The processed bacterial preparations were then used to produce two bivalent nanoemulsion vaccine formulations for the sequential immersion vaccination strategy: (i) *F. covae* plus *V. harveyi* for the primary immersion vaccination and (ii) *V. vulnificus* plus *P. damselae* for the secondary immersion booster. The cationic nanoemulsion system was prepared according to the optimized procedure described by Uchuwittayakul et al. (2025) [19] with minor modifications. Briefly, the formulation contained 86.8% (*w*/*w*) sonicated formalin-inactivated bacterial antigen at 1 × 10^12^ CFU/mL, 1.5% (*w*/*w*) polysorbate 80, Tween 80 (Merck Pte. Ltd., Singapore, Singapore) as a nonionic surfactant, 0.2% (*w*/*w*) cetyltrimethylammonium bromide, CTAB (Merck Pte. Ltd., Singapore, Singapore) as a cationic surfactant, 1.5% (*w*/*w*) sorbitan monooleate, Span 80 (Merck Pte. Ltd., Singapore, Singapore) and 10% (*w*/*w*) medium-chain triglycerides, MCTs (Tulip Chemplast Co., Ltd., Bangkok, Thailand) as the oil phase. The formalin-inactivated antigens were incorporated into the oil-in-water nanoemulsion by initial mixing at 400× *g* for 10 min, followed by high-shear homogenization at 35% amplitude for 20 min under chilled conditions at 4 °C, using a 1 min on and 1 min off pulse cycle. The resulting antigen-loaded cationic nanoemulsions were used for immersion vaccination.

The physicochemical characteristics of the nanoemulsion vaccines, including mean particle size, zeta potential, and polydispersity index (PDI), were evaluated using dynamic light scattering (DLS) with a Zetasizer Ultra instrument (Malvern Panalytical Ltd., Malvern, UK), following the manufacturer’s guidelines. Each formulation was analyzed in triplicate. The *F. covae* plus *V. harveyi* nanoemulsion formulation showed a mean particle size of 161.21 ± 1.33 nm, a PDI of 0.36 ± 0.01, and a positive zeta potential of 25.24 ± 0.28 mV. The *V. vulnificus* plus *P. damselae* formulation exhibited a mean particle size of 174.61 ± 1.04 nm, a PDI of 0.39 ± 0.01, and a positive zeta potential of 26.11 ± 0.35 mV. After preparation, the nanovaccine formulations were maintained at 4 °C. Their physicochemical parameters were reassessed immediately before each vaccination to verify formulation stability during the experimental period.

For oral immunization, a multivalent hydrogel vaccine containing *Fc*, *Vh*, *Vv*, and *Pd* antigens was formulated by entrapping equal amounts of the four formalin-inactivated bacterial preparations within a biodegradable mucoadhesive chitosan–alginate composite matrix. Briefly, the inactivated bacterial cells were suspended in deionized water to obtain a final concentration of 1 × 10^12^ CFU/mL, corresponding to a 5% (*w*/*v*) bacterial suspension. Calcium bentonite was then incorporated at a final concentration of 0.2% (*w*/*v*), followed by the addition of medium-viscosity sodium alginate, 200 to 400 cP, 2% (*w*/*v*) in water at 25 °C, to achieve a final concentration of 2% (*w*/*v*). The mixture was homogenized at 8000 rpm to ensure uniform antigen distribution. The resulting suspension was extruded dropwise through an 18G needle into a 3% (*w*/*v*) calcium chloride solution while stirring at 200 rpm. The droplets were allowed to crosslink for 30 min to form calcium alginate hydrogel beads. The beads were subsequently coated with 1% (*w*/*v*) chitosan (Sigma-Aldrich, Cat. No. 448869; molecular weight 50,000 to 190,000 Da; degree of deacetylation 75 to 85%) dissolved in 0.1 M acetic acid by stirring at 200 rpm for 45 min. The resulting chitosan-coated alginate hydrogel vaccine slurry was frozen at −80 °C for 24 h and then lyophilized at −65 °C for 24 h under a vacuum pressure of 4 mTorr to obtain a dry, stable vaccine powder. The lyophilized hydrogel vaccine was stored at 4 °C until further use.

### 4.3. Ethical Statement

This study adhered to the National Research Council of Thailand’s guidelines for animal use and received approval from the Kasetsart University Animal Ethics Committee (Approval No. ACKU68-FIS-037, 28 November 2025). To ensure welfare, all fish were anesthetized during handling and sample collection.

### 4.4. Fish Preparation and Husbandry

Juvenile Asian seabass were sourced from a commercial hatchery in Samut Sakhon Province, Thailand. Upon arrival, the fish were acclimatized for 14 days in 3000 L aerated fiberglass tanks containing dechlorinated freshwater adjusted to 10 ppt salinity. Water quality was maintained at 28 ± 2 °C, pH 7.2 ± 0.5, and dissolved oxygen above 6 mg/L throughout the acclimation period. Fish were fed a commercial diet containing 45% protein and 8% lipid twice daily at 5% of body weight per day. Before vaccination, the health status of the fish was assessed by bacterial culture and microscopic examination to confirm the absence of bacterial and parasitic infections.

At the beginning of the experiment, fish with an average body weight of 0.6 ± 0.1 g were randomly distributed into control and vaccinated groups. Each treatment consisted of three replicate tanks, with 50 fish per tank, giving a total of 150 fish per group. All fish were maintained in water at 10 ppt salinity during the experimental period.

### 4.5. Experimental Vaccination Protocol

Fish received a sequential program beginning with nanoemulsion immersion and progressing to oral hydrogel boosters.

- Dose 1 (immersion prime): fish were immersed for 60 min in a cationic nanoemulsion containing *F. covae* and *V. harveyi* at a 1:100 (*v*/*v*) vaccine-to-water ratio under continuous aeration.
- Dose 2 (immersion boost): 14 days later, fish were immersed using the same conditions with a nanoemulsion containing *V. vulnificus* and *P. damselae*.
- Dose 3 (oral hydrogel): 14 days after Dose 2, a multivalent hydrogel (*Fc, Vh, Vv, Pd*) was top-dressed onto feed at 10% *w*/*w* of the daily ration and administered for 7 consecutive days. Routine feeding was twice daily at 5% body weight per day.
- Dose 4 (oral booster): 30 days after Dose 3, the same hydrogel vaccine was provided for 5 consecutive days at the same inclusion rate.
- Dose 5 (final oral booster): 30 days after Dose 4, the hydrogel vaccine was given for 3 consecutive days at the same inclusion rate.

The control fish were maintained under the same husbandry conditions and received the same basal diet without vaccine. Blank nanoemulsion and blank hydrogel vehicle groups were not included.

### 4.6. Bacterial Challenge Procedures

Challenges were performed 30 days after Dose 5. Vaccinated and control fish were exposed under pathogen-specific conditions using doses calibrated to the previously established 14-day LD_50_ or LC_50_ for each pathogen.

1. *Fc* immersion (0 ppt): fish were immersed for 2 h in freshwater (0 ppt) containing *Flavobacterium covae* at 1 × 10^5^ CFU mL^−1^ with continuous aeration.
2. *Vh/Vv/Pd* immersion (10 ppt): fish were immersed for 2 h in 10-ppt water containing a mixed suspension of *Vibrio harveyi*, *Vibrio vulnificus*, and *Photobacterium damselae* at a total of 1 × 10^6^ CFU mL^−1^ (equal parts of each species) under continuous aeration.
3. *Vh/Vv/Pd* injection (10 ppt): fish received an intraperitoneal injection of 0.1 mL per fish of the mixed suspension at 1 × 10^6^ CFU mL^−1^ total (3.33 × 10^5^ CFU mL^−1^ of each species), delivering 1 × 10^5^ CFU per fish in total (≈3.33 × 10^4^ CFU of each pathogen).

The viable bacterial concentrations used for challenge were confirmed by standard plate-count enumeration. Each bacterial suspension was serially diluted and plated onto the appropriate agar medium. After incubation under the corresponding growth conditions, colonies were counted and expressed as CFU mL^−1^. The suspensions were then adjusted to the required challenge concentrations.

At 24 h post-challenge, three surviving fish per treatment were selected for omics analysis. Each biological replicate consisted of tissue from one individual fish, and samples were not pooled. The three fish were obtained from three different replicate tanks. Tank identity was recorded during sampling. Thus, *n* = 3 represents three individual biological samples per treatment for each tissue per challenge comparison, rather than technical replicates. The same three-sample biological replicate structure was used for RNA-seq and label-free proteomics. For immersion challenges, gills were collected; for injection challenges, intestine was collected to assess mucosal responses. Tissues were processed immediately for RNA using the easy-spin™ Total RNA Extraction Kit (iNtRON Biotechnology, Seongnam, Republic of Korea) and stored at −80 °C pending sequencing.

For each challenge model, survival was assessed using 30 fish per treatment, comprising three replicate tanks with 10 fish per tank. Fish were monitored for 14 days after challenge, and survival distributions were estimated using the Kaplan–Meier method. Survival curves between the vaccinated and control groups were compared using the log-rank (Mantel–Cox) test, with statistical significance defined as *p* < 0.05. Relative percent survival (RPS) was calculated as RPS = [1 − (% mortality in vaccinated fish/% mortality in control fish)] × 100. Survival analyses and graphical visualization were performed using GraphPad Prism version 10.4.0 (GraphPad Software, Boston, MA, USA).

### 4.7. Transcriptome Profiling of Mucosal Tissues

#### 4.7.1. Total RNA Isolation, Quantification and Qualification

Total RNA was extracted from gill and intestinal tissues using the easy-spin™ Total RNA Extraction Kit (iNtRON Biotechnology, Seongnam, Republic of Korea) according to the manufacturer’s instructions. RNA concentration and purity were measured with a NanoDrop 2000 spectrophotometer (Thermo Fisher Scientific, Wilmington, DE, USA). Integrity was first screened by electrophoresis on a 1% agarose gel and then verified with an Agilent 2100 Bioanalyzer (Agilent Technologies, Palo Alto, CA, USA).

#### 4.7.2. Library Construction and Sequencing and Quality Control

For each sample, 1 μg of total RNA was used to construct libraries with the NEBNext Ultra RNA Library Prep Kit for Illumina (New England Biolabs, Ipswich, MA, USA) following the manufacturer’s instructions. mRNA was captured with poly-T oligo-magnetic beads, fragmented, and reverse-transcribed using random hexamers and M-MuLV Reverse Transcriptase, followed by second-strand synthesis with DNA Polymerase I. The resulting cDNA was purified, end-repaired, adenylated, ligated to indexed adapters, and size-selected to 300–400 bp with AMPure XP beads AMPure XP beads (Beckman Coulter, Brea, CA, USA). Library concentration and size distribution were verified by Qubit 2.0 fluorometry, Agilent 2100 Bioanalyzer, and qPCR; only libraries exceeding 2 nM proceeded to sequencing. High-quality libraries were sequenced by Biomarker Technologies (Münster, Germany) on an Illumina NovaSeq 4000 platform in paired-end 150 bp mode. Downstream analyses used the Asian seabass reference genome (BioSample SAMN04026617; NCBI RefSeq GenBank GCF_001640805.2).

Adapters, low-quality reads, and poly-N sequences were trimmed to produce clean reads; Q20/Q30 and GC content were recorded. Clean reads were aligned to the reference genome using HISAT2, and reads with ≥2 mismatches were discarded, retaining only perfect or one-mismatch alignments.

#### 4.7.3. Gene Functional Annotation

Functional annotation integrated complementary resources: broad sequence identity from NR (NCBI, https://www.ncbi.nlm.nih.gov, accessed on 2 August 2025) and Swiss-Prot (UniProt, https://www.uniprot.org, accessed on 2 August 2025); domains/families from Pfam (https://pfam.xfam.org, accessed on 2 August 2025); orthology classification with COG and KOG (NCBI, https://www.ncbi.nlm.nih.gov, accessed on 2 August 2025); GO term mapping via Gene Ontology (https://geneontology.org, accessed on 2 August 2025); pathway assignment from KEGG (https://www.genome.jp/kegg/, accessed on 2 August 2025); interaction context using STRING (https://string-db.org, accessed on 2 August 2025); gene models/IDs reconciled through Ensembl (https://www.ensembl.org, accessed on 2 August 2025).

#### 4.7.4. Sample Relationship and Differential Expression Analysis

Principal component analysis (PCA) was performed in R (https://www.r-project.org, accessed on 10 August 2025) to assess replicate consistency and screen for potential outliers. Differential expression was analyzed with DESeq2, using only comparisons with biological replicates. Genes were considered differentially expressed at FDR-adjusted *p* < 0.05 and absolute fold change > 1.5, and these same thresholds were applied for subsequent enrichment tests. Gene Ontology (GO) enrichment analysis was performed using GOseq, which accounts for gene-length bias, whereas Kyoto Encyclopedia of Genes and Genomes (KEGG) pathway enrichment was conducted using KOBAS. Differentially expressed genes were used as the query set, and genes detected and successfully annotated in the corresponding transcriptomic dataset were used as the background set. Enrichment significance was evaluated using multiple-testing-adjusted *p* values, with FDR-adjusted *p* < 0.05 considered statistically significant. For each enriched GO term and KEGG pathway, the number of DEGs assigned to the term or pathway, the gene ratio, the corresponding background gene number, and the FDR-adjusted *p* value were recorded. The gene ratio was defined as the number of DEGs assigned to a given term or pathway divided by the total number of DEGs included in the enrichment analysis. Protein–protein interaction networks were inferred by querying DEG identifiers against STRING v12.0 (https://string-db.org, accessed on 10 August 2025), and network visualization was carried out using Cytoscape v3.10.4 (https://www.cytoscape.org, accessed on 10 August 2025).

#### 4.7.5. Validation of Expression Profiles Using Real-Time Quantitative PCR (RT-qPCR)

Ten DEGs, including both up- and downregulated transcripts, were selected from the most dominant and group-specific candidates for validation by real-time quantitative PCR (RT-qPCR). For each sample, 1000 ng total RNA was reverse-transcribed using Maxime™ RT PreMix (iNtRON Biotechnology, Seongnam, Republic of Korea) according to the manufacturer’s instructions. First-strand cDNA was stored at −20 °C until use. Reactions were prepared with Brilliant III Ultra-Fast SYBR^®^ Green (Agilent Technologies, Santa Clara, CA, USA) and run on an AriaMx Real-Time PCR System (Agilent, CA, USA). Cycling parameters were: 95 °C for 5 min; 40 cycles of 95 °C for 30 s, 60 °C for 30 s, and 72 °C for 90 s, followed by a final extension at 72 °C for 10 min.

Primer specificity was confirmed by the presence of a single peak in melt-curve analysis. Amplification efficiencies were determined from standard curves generated using serial dilutions of pooled cDNA and were calculated from the slope of each standard curve as *E* = 10^(−1/slope)^ − 1, with optimal efficiency falling between 0.90 and 1.10 (90–110%). The reference genes *actb*, *gapdh*, and *ef1a* were selected based on their previous validation and use in Asian seabass and their stable C_t_ values across the experimental samples examined in the present study. For each sample, the normalization factor was calculated as the geometric mean of the relative expression values of the three reference genes. Relative expression was calculated using the 2^−ΔΔCt^ method and reported as log2 fold change. All primer pairs were validated for specific amplification and performance prior to experiments. Primer sequences for all targets and references are listed in Table S1.

Genes selected for RT-qPCR validation were chosen based on the magnitude or group specificity of their RNA-seq responses, biological relevance to the major enriched immune and cellular pathways, and representation of both upregulated and downregulated transcripts.

#### 4.7.6. Label-Free Proteomic Analysis

Whole-protein lysates were prepared from gill and intestinal tissues of three fish per group. Each biological replicate consisted of tissue collected from one individual fish, and samples were not pooled. The three fish were obtained from three different replicate tanks, and tank identity was recorded during sampling. Thus, *n* = 3 represented three independent biological samples per treatment. Tissue lysates were prepared in radioimmunoprecipitation assay (RIPA) buffer, sonicated on ice (3 s on/3 s off for 10 min), and clarified by centrifugation at 12,000× *g* for 10 min at 4 °C. Protein concentration was measured by the bicinchoninic acid assay (BCA). Aliquots (50 µg) were reduced with tris(2-carboxyethyl)phosphine (TCEP) at 56 °C for 1 h and alkylated with iodoacetamide (IAA) in the dark at room temperature (RT) for 40 min. Proteins were digested overnight with sequencing-grade trypsin (37 °C). Peptides were desalted using single-pot, solid-phase cleanup (SP2) with paramagnetic beads (binding in 95% acetonitrile (ACN); triple ACN washes; elution in 2% ACN), dried, and reconstituted prior to liquid chromatography–tandem mass spectrometry (LC–MS/MS).

Peptides were separated on a nano-LC system using a C18 trap (75 µm × 2 cm, 3 µm) and analytical column (150 µm inner diameter (i.d.) × 170 mm, 1.9 µm) at 300 nL/min with a 131 min gradient (4–28% solvent B over 105 min, then to 40% at 120 min and 95% at 121–131 min; solvent A: 0.1% formic acid (FA) in water; solvent B: 80% ACN/0.1% FA). Mass spectra were acquired on an Orbitrap Exploris 480 mass spectrometer (Thermo Fisher Scientific, Bremen, Germany) in data-dependent acquisition (DDA) mode: MS1 resolution 120,000 (*m*/*z* 300–1800), maximum injection time (max IT) 20 ms; MS2 resolution 15,000, max IT 22 ms, cycle time 1.3 s, normalized collision energy (NCE) 30.

Raw LC-MS/MS data were processed using MaxQuant v1.5.2.8 against the *Lates calcarifer* UniProt reference proteome. Peptide-spectrum matches and protein identifications were controlled at a 1% false discovery rate (FDR) using the target-decoy strategy implemented in MaxQuant. Proteins supported by at least one unique peptide were retained for quantitative analysis. Label-free quantification was performed at the protein level using normalized LFQ intensities. Between-group differences were evaluated using Student’s *t*-test based on three independent biological replicates per treatment. Protein-level *p* values were adjusted for multiple testing using the Benjamini–Hochberg procedure, and proteins with FDR-adjusted *p* < 0.05 were considered statistically significant.

## 5. Conclusions

This study provides a systems-level view of molecular responses associated with sequential nanoemulsion immersion and oral hydrogel vaccination in Asian seabass challenged with *F. covae*, *V. harveyi*, *V. vulnificus*, and *P. damselae*. Transcriptomic and proteomic analyses identified coordinated signatures related to innate and humoral immunity, leukocyte trafficking, complement, lysosomal activity, and epithelial responses, while RT-qPCR supported selected transcriptomic changes. These molecular responses were accompanied by improved survival following bacterial challenge. However, different challenge routes were used for gill and intestinal sampling, and antigen-specific antibodies, bacterial load, histological protection, functional immune responses, targeted protein validation, and quantitative transcript–protein integration were not assessed. Therefore, the identified pathways should be interpreted as vaccination-associated molecular signatures rather than direct evidence of functional immune mechanisms. Overall, these findings provide candidate markers and a framework for further development of multivalent mucosal vaccines in Asian seabass.

## Data Availability

The original contributions presented in this study are included in the article. Further inquiries can be directed to the corresponding author.

## Supplementary Materials

The following supporting information can be downloaded at https://www.mdpi.com/article/10.3390/ijms27177652/s1.

## References

[1] Veedu A.K., Thomas J. (2026). Probiotics and prebiotics as alternatives to antibiotics in aquaculture: A systematic and bibliometric review of antimicrobial and antioxidant mechanisms. Front. Microbiol..

[2] Basili M., Nikouli E., Møller F.D., Kormas K.A., Luna G.M., Tolosa-Enguís V., Rodriguez-Ruano S.M., Sanz Y., Candela M., Quero G.M. (2026). Aquaculture shapes antibiotic resistance in Mediterranean gilthead sea bream farming: From hatcheries to offshore cages. Aquaculture.

[3] Schar D., Zhao C., Wang Y., Larsson D.G.J., Gilbert M., Van Boeckel T.P. (2021). Twenty-year trends in antimicrobial resistance from aquaculture and fisheries in Asia. Nat. Commun..

[4] Pepi M., Focardi S. (2021). Antibiotic-Resistant Bacteria in Aquaculture and Climate Change: A Challenge for Health in the Mediterranean Area. Int. J. Environ. Res. Public Health.

[5] Schar D., Klein E.Y., Laxminarayan R., Gilbert M., Van Boeckel T.P. (2020). Global trends in antimicrobial use in aquaculture. Sci. Rep..

[6] Munoz-Atienza E., Diaz-Rosales P., Tafalla C. (2020). Systemic and Mucosal B and T Cell Responses Upon Mucosal Vaccination of Teleost Fish. Front. Immunol..

[7] Mondal H., Thomas J. (2022). A review on the recent advances and application of vaccines against fish pathogens in aquaculture. Aquac. Int..

[8] Rathor G.S., Swain B. (2024). Advancements in Fish Vaccination: Current Innovations and Future Horizons in Aquaculture Health Management. Appl. Sci..

[9] Du Y., Hu X., Miao L., Chen J. (2022). Current status and development prospects of aquatic vaccines. Front. Immunol..

[10] Kumar A., Middha S.K., Menon S.V., Paital B., Gokarn S., Nelli M., Rajanikanth R.B., Chandra H.M., Mugunthan S.P., Kantwa S.M. (2024). Current Challenges of Vaccination in Fish Health Management. Animals.

[11] Huang Y., Yang X., Liu W., Wang P., Huang M. (2026). Advances in aquatic immersion vaccines: Technological evolution, strategies for overcoming mucosal barriers, and future prospects. Aquac. Int..

[12] Yu Y., Wang Q., Huang Z., Ding L., Xu Z. (2020). Immunoglobulins, Mucosal Immunity and Vaccination in Teleost Fish. Front. Immunol..

[13] Somamoto T., Nakanishi T. (2020). Mucosal delivery of fish vaccines: Local and systemic immunity following mucosal immunisations. Fish Shellfish Immunol..

[14] Cao Y., Liu J., Liu G., Du H., Liu T., Wang G., Wang Q., Zhou Y., Wang E. (2023). Exploring the Immunoprotective Potential of a Nanocarrier Immersion Vaccine Encoding Sip against *Streptococcus* Infection in Tilapia (*Oreochromis niloticus*). Vaccines.

[15] Zhu B., Zhang C., Zhao Z., Wang G.X. (2020). Targeted Delivery of Mannosylated Nanoparticles Improve Prophylactic Efficacy of Immersion Vaccine against Fish Viral Disease. Vaccines.

[16] Zhao K., Xie Y., Lin X., Xu W. (2022). The Mucoadhesive Nanoparticle-Based Delivery System in the Development of Mucosal Vaccines. Int. J. Nanomed..

[17] Bashir S., Phuoc N.N., Herath T., Basit A., Zadoks R.N., Murdan S. (2023). An oral pH-responsive *Streptococcus agalactiae* vaccine formulation provides protective immunity to pathogen challenge in tilapia: A proof-of-concept study. PLoS ONE.

[18] Wang E., Wang J., Long B., Wang K., He Y., Yang Q., Chen D., Geng Y., Huang X., Ouyang P. (2018). Preparation, characterization and evaluation of the immune effect of alginate/chitosan composite microspheres encapsulating recombinant protein of *Streptococcus iniae* designed for oral vaccination. Fish Shellfish Immunol..

[19] Uchuwittayakul A., Thompson K.D., Thangsunan P., Phaksopa J., Buncharoen W., Saenphet K., Kumwan B., Meachasompop P., Saenphet S., Wiratama N. (2025). Evaluation of a hydrogel platform for encapsulated multivalent *Vibrio* antigen delivery to enhance immune responses and disease protection against vibriosis in Asian seabass (*Lates calcarifer*). Fish Shellfish Immunol..

[20] Lan N.G.T., Dong H.T., Vinh N.T., Senapin S., Shinn A.P., Salin K.R., Rodkhum C. (2024). Immersion prime and oral boost vaccination with an inactivated *Vibrio harveyi* vaccine confers a specific immune response and protection in Asian seabass (*Lates calcarifer*). Fish Shellfish Immunol..

[21] Vanichavetin K., Uchuwittayakul A., Namdee K., Srisapoome P. (2024). Oral booster effects of bivalent nanovaccine-primed fingerlings of Asian seabass (*Lates calcarifer*, Bloch 1790) to prevent streptococcosis and columnaris diseases. Aquaculture.

[22] Rodwihok C., Thompson K.D., Srisapoome P., Thangsunan P., Thangsunan P., Buncharoen W., Saenphet K., Saenphet S., Meachasompop P., Kumwan B. (2025). Evaluation of immune responses and protection in Asian seabass (*Lates calcarifer* Bloch, 1790) against *Vibrio vulnificus* using immersion and oral nanoemulsion vaccines. Fish Shellfish Immunol..

[23] Salinas I., Fernandez-Montero A., Ding Y., Sunyer J.O. (2021). Mucosal immunoglobulins of teleost fish: A decade of advances. Dev. Comp. Immunol..

[24] Xu Z., Takizawa F., Casadei E., Shibasaki Y., Ding Y., Sauters T.J.C., Yu Y., Salinas I., Sunyer J.O. (2020). Specialization of mucosal immunoglobulins in pathogen control and microbiota homeostasis occurred early in vertebrate evolution. Sci. Immunol..

[25] Uchuwittayakul A., Thangsunan P., Thangsunan P., Rodkhum C., Srisapoome P. (2024). Molecular structure and functional responses of IgM, IgT and IgD to *Flavobacterium covae* and *Streptococcus iniae* infection in Asian seabass (*Lates calcarifer* Bloch, 1790). Fish Shellfish Immunol..

[26] Hopo M.G., Mabrok M., Abu-Elala N., Yu Y. (2024). Navigating Fish Immunity: Focus on Mucosal Immunity and the Evolving Landscape of Mucosal Vaccines. Biology.

[27] Zhang Z., Hong W., Zhang Y., Li X., Que H., Wei X. (2025). Mucosal immunity and vaccination strategies: Current insights and future perspectives. Mol. Biomed..

[28] Li J.-N., Zhao Y.-T., Cao S.-L., Wang H., Zhang J.-J. (2020). Integrated transcriptomic and proteomic analyses of grass carp intestines after vaccination with a double-targeted DNA vaccine of *Vibrio mimicus*. Fish Shellfish Immunol..

[29] Cao M., Yan X., Su B., Yang N., Fu Q., Xue T., Song L., Li Q., Li C. (2020). Integrated Analysis of circRNA-miRNA-mRNA Regulatory Networks in the Intestine of *Sebastes schlegelii* Following *Edwardsiella tarda* Challenge. Front. Immunol..

[30] Silvaraj S., Yasin I.S.M., Karim M.M.A., Saad M.Z. (2021). Transcriptome analysis of immune response in recombinant cell vaccine expressing OmpK vaccinated juvenile seabass (*Lates calcarifer*) head kidney against *Vibrio harveyi* infection. Aquac. Rep..

[31] Raju T., Manchanayake T., Danial A., Zamri-Saad M., Azmai M.N.A., Md Yasin I.S., Mohd Nor N., Salleh A. (2023). Evaluating the Intestinal Immunity of Asian Seabass (*Lates calcarifer*, Bloch 1790) following Field Vaccination Using a Feed-Based Oral Vaccine. Vaccines.

[32] Porter D., Collins C., Mazzolini A., Jouneau L., Mhanna V., Coiffier C., Peruzzi M., Jaszczyszyn Y., Mariotti-Ferrandiz E., Huetz F. (2025). Divergent B-cell repertoire remodelling by mRNA, DNA and live attenuated vaccines in fish. npj Vaccines.

[33] Attaya A., Secombes C.J., Wang T. (2020). Effective isolation of GALT cells: Insights into the intestine immune response of rainbow trout (*Oncorhynchus mykiss*) to different bacterin vaccine preparations. Fish Shellfish Immunol..

[34] Tafalla C. (2024). Understanding fish B cell responses to combat infectious diseases. Bull. Eur. Assoc. Fish Pathol..

[35] Semple S.L., Dixon B. (2020). Salmonid Antibacterial Immunity: An Aquaculture Perspective. Biology.

[36] FAO *Lates calcarifer* (Bloch, 1790). Cultured Aquatic Species Information Programme. Fisheries and Aquaculture Division, Food and Agriculture Organization of the United Nations, Rome, Italy. https://www.fao.org/fishery/en/culturedspecies/lates_calcarifer.

[37] Nhan D.T., Tu N.P.C., Tu N.V. (2022). Comparison of growth performance, survival rate and economic efficiency of Asian seabass (*Lates calcarifer*) intensively cultured in earthen ponds with high densities. Aquaculture.

[38] Yue G.H. (2025). Asian Seabass (*Lates calcarifer*): A Versatile Species for Marine and Freshwater Aquaculture. Rev. Aquac..

[39] Muangrerk C., Uchuwittayakul A., Srisapoome P. (2024). Identification, Expression and Antimicrobial Functional Analysis of Interleukin-8 (IL-8) in Response to *Streptococcus iniae* and *Flavobacterium covae* in Asian Seabass (*Lates calcarifer* Bloch, 1790). Animals.

[40] Raharjo H.M., Budiyansah H., Mursalim M.F., Chokmangmeepisarn P., Sakulworakan R., Madyod S., Sewaka M., Sonthi M., Debnath P.P., Elayaraja S. (2022). Distribution of Vibrionaceae in farmed Asian sea bass, *Lates calcarifer* in Thailand and their high prevalence of antimicrobial resistance. J. Fish Dis..

[41] Erfanmanesh A., Beikzadeh B., Khanzadeh M., Adel M., Gholamhosseini A., Soltani M. (2024). Immuno-protective response of Asian seabass (*Lates calcarifer*) to inactivated vaccines against *Streptococcus iniae* and *Vibrio harveyi*. BMC Vet. Res..

[42] Weawsawang W., Homsombat T., Nuanmanee S., Saleetid N., Thawonsuwan J., Pumchan A., Hirono I., Kondo H., Unajak S. (2024). Characterization of *Photobacterium damselae* subsp. damselae isolated from diseased Asian seabass (*Lates calcarifer*) and the preliminary development of a formalin-killed cell vaccine. J. Fish Dis..

[43] Nguyen D.H.M., Chokmangmeepisarn P., Khianchaikhan K., Morishita M., Uchuwittayakul A., LaFrentz B.R., Rodkhum C. (2025). Comparative genomic analysis of *Flavobacterium* species causing columnaris disease of freshwater fish in Thailand: Insights into virulence and resistance mechanisms. BMC Vet. Res..

[44] Yue G., Guo C. (2025). Strategies for managing major diseases in Asian seabass aquaculture. Anim. Dis..

[45] Lan N.G.T., Dong H.T., Vinh N.T., Senapin S. (2025). Non-invasive vaccination enhances immune response and protects Asian seabass (*Lates calcarifer*) against *Vibrio vulnificus* infection. Comp. Immunol. Rep..

[46] Chan J., Carmen L.C.P., Lee S.Q., Prabakaran M. (2023). Identification and characterization of immunoglobulin tau (IgT) in Asian Seabass (*Lates calcarifer*) and mucosal immune response to nervous necrosis virus. Front. Immunol..

[47] Bobrovskikh A.V., Zubairova U.S., Doroshkov A.V. (2023). Fishing Innate Immune System Properties through the Transcriptomic Single-Cell Data of Teleostei. Biology.

[48] Loh Z., Lim T.W., Howland S.W., Awate S., Renia L., Chen J., Ren E.C. (2024). Single-Cell Transcriptome Profiling of Scale Drop Disease Virus-Infected Asian Seabass (*Lates calcarifer*). Aquac. J..

[49] Wang X., Gao Y., Ni X., Guo Z., Zhang J., Wang X., Li R. (2024). Transcriptomic analysis of gene expression in immune pathways in the spleen of *Takifugu rubripes* after immunization with scuticociliate vaccine. Aquaculture.

[50] Gao C., Cai X., Lymbery A.J., Ma L., Cao M., Li C. (2023). Integrative transcriptomic profiling reveals the key pathways in the regulation mechanism of fish intestine-spleen immunity during the bacterial challenges. Aquaculture.

[51] Goh C., Gibson-Kueh S., Bal D., Chen I.T., Nuez-Ortín W., Domingos J.A., Shen X. (2025). A synergistic blend of dietary organic acids, monoglycerides and phytobiotics enhance growth performance, intestinal mucosal height, and anti-viral immune gene expression in juvenile Barramundi (*Lates calcarifer*). Aquac. Rep..

[52] Tian S., Chen P., Wu Z., Wu Y., Yuan J., Huang D., Mai K., Zhang W. (2024). Dietary cottonseed protein concentrate affected the flesh texture and myofiber characteristics of large yellow croaker *Larimichthys crocea*. Aquaculture.

[53] Ghani M.U., Chen J., Khosravi Z., Wu Q., Liu Y., Zhou J., Zhong L., Cui H. (2024). Unveiling the multifaceted role of toll-like receptors in immunity of aquatic animals: Pioneering strategies for disease management. Front. Immunol..

[54] Sathyan K.R., Premraj A., Puthiyedathu S.T. (2024). Molecular characterisation and expression analysis of an interferon-induced protein with tetratricopeptide repeats 1 (IFIT1) homologue from Asian seabass (*Lates calcarifer*). Comp. Immunol. Rep..

[55] Krishna Priya R.S., Premraj A., Sivakumar K.C., Sajeevan T.P. (2022). Identification of two ISG15 homologues involved in host immune response against RGNNV in Asian seabass (*Lates calcarifer*). Fish Shellfish Immunol. Rep..

[56] Arbanasić H., Medrano-González L., Hrenar T., Mikelić A., Gomerčić T., Svetličić I., Pavlinec Ž., Đuras M., Galov A. (2024). Recent selection created distinctive variability patterns on MHC class II loci in three dolphin species from the Mediterranean Sea. Dev. Comp. Immunol..

[57] Domingos J.A., Shen X., Terence C., Senapin S., Dong H.T., Tan M.R., Gibson-Kueh S., Jerry D.R. (2021). Scale Drop Disease Virus (SDDV) and *Lates calcarifer* Herpes Virus (LCHV) Coinfection Downregulate Immune-Relevant Pathways and Cause Splenic and Kidney Necrosis in Barramundi Under Commercial Farming Conditions. Front. Genet..

[58] Sutra J., Hashim A.M., Yusof M.T., Al-Saari N., Nasruddin N.S., Saad M.Z., Zahaludin A.-D., Yasin I.S.M., Azmai M.N.A. (2025). Dynamics of the gut microbiome of Asian seabass (*Lates calcarifer*) following oral vaccination and challenge with virulent *Vibrio harveyi*. Aquaculture.

[59] Zhang Y.-A., Salinas I., Li J., Parra D., Bjork S., Xu Z., LaPatra S.E., Bartholomew J., Sunyer J.O. (2010). IgT, a primitive immunoglobulin class specialized in mucosal immunity. Nat. Immunol..

[60] Thu Lan N.G., Salin K.R., Longyant S., Senapin S., Dong H.T. (2021). Systemic and mucosal antibody response of freshwater cultured Asian seabass ((*Lates calcarifer*, Bloch 1790)) to monovalent and bivalent vaccines against *Streptococcus agalactiae* and *Streptococcus iniae*. Fish Shellfish Immunol..

[61] Vinh N.T., Dong H.T., Lan N.G.T., Sangsuriya P., Salin K.R., Chatchaiphan S., Senapin S. (2023). Immunological response of 35 and 42 days old Asian seabass (*Lates calcarifer*, Bloch 1790) fry following immersion immunization with *Streptococcus iniae* heat-killed vaccine. Fish Shellfish Immunol..

[62] Chokmangmeepisarn P., Senapin S., Taengphu S., Thompson K.D., Srisapoome P., Uchuwittayakul A., Rodkhum C. (2024). Protective efficiency and immune responses to single and booster doses of formalin-inactivated scale drop disease virus (SDDV) vaccine in Asian seabass (*Lates calcarifer*). BMC Vet. Res..

[63] Tanpichai P., Chaweepack S., Senapin S., Piamsomboon P., Wongtavatchai J. (2023). Immune Activation Following Vaccination of *Streptococcus iniae* Bacterin in Asian Seabass (*Lates calcarifer*, Bloch 1790). Vaccines.

[64] Sullivan R., Becker J.A., Samsing F. (2025). Integrative analysis of the microRNA and mRNA response of barramundi (*Lates calcarifer*) under acute cold stress and *Vibrio harveyi* challenge. Dev. Comp. Immunol..

[65] Guerra-Espinosa C., Jimenez-Fernandez M., Sanchez-Madrid F., Serrador J.M. (2024). ICAMs in Immunity, Intercellular Adhesion and Communication. Cells.

[66] Zapata A.G. (2022). Lympho-Hematopoietic Microenvironments and Fish Immune System. Biology.

[67] Chan J.T.H., Kadri S., Kollner B., Rebl A., Korytar T. (2022). RNA-Seq of Single Fish Cells—Seeking Out the Leukocytes Mediating Immunity in Teleost Fishes. Front. Immunol..

[68] Cao J., Xu H., Yu Y., Xu Z. (2023). Regulatory roles of cytokines in T and B lymphocytes-mediated immunity in teleost fish. Dev. Comp. Immunol..

[69] Lu T.Z., Liu X., Wu C.S., Ma Z.Y., Wang Y., Zhang Y.A., Zhang X.J. (2022). Molecular and Functional Analyses of the Primordial Costimulatory Molecule CD80/86 and Its Receptors CD28 and CD152 (CTLA-4) in a Teleost Fish. Front. Immunol..

[70] Tian H.F., Xing J., Tang X.Q., Chi H., Sheng X.Z., Zhan W.B. (2022). Cluster of differentiation antigens: Essential roles in the identification of teleost fish T lymphocytes. Mar. Life Sci. Technol..

[71] Xing J., Hu Y., Liu W., Tang X., Sheng X., Chi H., Zhan W. (2024). The interaction between the costimulatory molecules CD80/86 and CD28 contributed to CD4(+) T lymphocyte activation in flounder (*Paralichthys olivaceus*). Fish Shellfish Immunol..

[72] Zou J., Mustafa N., Wang J. (2026). Fish CD8+ T cells: Current knowledge and research perspectives. Water Biol. Secur..

[73] Abos B., Wang T., Secombes C.J., Tafalla C. (2020). Distinct modes of action of CD40L and adaptive cytokines IL-2, IL-4/13, IL-10 and IL-21 on rainbow trout IgM(+) B cells. Dev. Comp. Immunol..

[74] Li B., Yang Y., Li Y., Wu L., Wu S., Dong Z., Chen M., Liang F., Guo Z., Wang B. (2021). CD40 plays roles in T cell-dependent response and B cell activation and differentiation in Nile tilapia (*Oreochromis niloticus*). Aquaculture.

[75] Herranz-Jusdado J.G., Morel E., Simon R., Diaz-Rosales P., Tafalla C. (2023). Teleost IgD(+)IgM(-) B cells in gills and skin have a plasmablast profile, but functionally and phenotypically differ from IgM(+)IgD(-) B cells in these sites. iScience.

[76] LaFrentz B.R., Kralova S., Burbick C.R., Alexander T.L., Phillips C.W., Griffin M.J., Waldbieser G.C., Garcia J.C., de Alexandre Sebastiao F., Soto E. (2022). The fish pathogen *Flavobacterium columnare* represents four distinct species: *Flavobacterium columnare*, *Flavobacterium covae* sp. nov., *Flavobacterium davisii* sp. nov. and *Flavobacterium oreochromis* sp. nov., and emended description of *Flavobacterium columnare*. Syst. Appl. Microbiol..

[77] Raharjo H.M., Budiyansah H., Mursalim M.F., Chokmangmeepisarn P., Sakulworakan R., Debnath P.P., Sivaramasamy E., Intan S.T., Chuanchuen R., Dong H.T. (2023). The first evidence of blaCTX-M-55, QnrVC5, and novel insight into the genome of MDR *Vibrio vulnificus* isolated from Asian sea bass (*Lates calcarifer*) identified by resistome analysis. Aquaculture.

[78] Lan N.G.T., Dong H.T., Vinh N.T., Senapin S., Shinn A.P., Salin K.R., Rodkhum C. (2024). Review of current perspectives and future outlook on bacterial disease prevention through vaccination in Asian seabass (*Lates calcarifer*). J. Fish Dis..

