# Supplementary material for: Transcriptomic and Proteomic Insights into Mucosal Immune Responses of Asian Seabass (Lates calcarifer) After Sequential Mucosal Vaccination Against Bacterial Pathogens
Source: Int J Mol Sci. 2026 Aug 26;27(17):7652. doi: 10.3390/ijms27177652 (PMC13566311; doi:10.3390/ijms27177652)
Supplement: Supplementary file 1 [file ijms-27-07652-s001.zip › ijms-4410905-supplementary.pdf]

Supplementary Document

Transcriptomic and proteomic signatures of mucosal immunity in Asian seabass following sequential nanoemulsion immersion and oral hydrogel vaccination against four key bacterial pathogens

Table S1. Primer sequences of target and reference genes used for RT-qPCR validation

Gene	Primer names	Nucleotide sequences (5′→3′)	Tm (°C)	Efficiency (%)	Reference
Macrophage mannose receptor 1 (CD206)	*Lc_mrc1*	F: ACCAACAGTTCCTCCAAAAGGT<br>R: TAGGAGCCTTGGCCATTTCAAT	60	96.4	XM_018676200.2
Interleukin-1 beta	*Lc_il1b*	F: TTCAGAGACGAACACCTGCTCA<br>R: GGTCGACATGTTCAGGTGTACT	60	101.7	XM_018669006.2
Interferon-induced GTP-binding protein MX1	*Lc_mx1*	F: GATAAGCTACCAGGACCACGAG<br>R: TGCCAGGCAGATCAATAAGTGT	60	94.8	XM_051074680.1
Immunoglobulin heavy constant mu (IgM)	*Lc_ighm*	F: AAGGTAAACGAGGGAGCTGTTG<br>R: TGGCTCCATGCCATCTACAAGT	60	103.2	In this study
Complement component 1, q subcomponent (C1q)	*Lc_c1qa*	F: AAGGAGGGAACACCGGAAATAC<br>R: CCACGAAGCCCAGACTTTATCT	60	98.6	XM_018666047.2
CXC chemokine receptor (generic)	*Lc_cxcr*	F: GATCGACACGCTCATAAAAGGC<br>R: CTAAGAGCAGCTGGTTCCTGAA	60	105.1	XM_018682251.2
Tumor necrosis factor receptor (superfamily)	*Lc_tnfrsf*	F: CAGCACAGAGACACAAACACTG<br>R: CTGGTCTGATCAGCTGAAGGTT	60	92.9	XM_018682821.2
Insulin-like growth factor 1 receptor	*Lc_igf1r*	F: TCACGACTCAGGGAGATCTTCT<br>R: TCACGACTCAGGGAGATCTTCT	60	100.4	XM_051076570.1
C3a chemokine receptor 1	*Lc_c3ar1*	F: ACATGTTCTGCTCCGTCTTTCT<br>R: GTTCTTCCCTGGGTAGACTTGG	60	97.3	XM_018682103.2
Complement C4	*Lc_c4*	F: TTGCTTCTTCCCTACAGTG<br>R: GGTCCAACCCTCCTTTAC	60	106.2	In this study
CC chemokine receptor (generic)	*Lc_ccr*	F: GACCCGGAACATAGCCTATCTG<br>R: TCTCTGTGATGTGGTTCTGGTG	60	95.6	XM_018695961.2
Class I histocompatibility antigen (MHC class I)	*Lc_mhc1*	F: GCAGTGGTTCAAGGTCAACATC<br>R: AAGTCGTTTCCATCGTAGCTGT	60	102.5	XM_018688353.2
Class II histocompatibility antigen (MHC class II)	*Lc_mhc2*	F: CCCAAACAGATCAGAGTGAGCT<br>R: CCCAGTCAGTAACCAGAGGTTC	60	99.1	XM_018674780.2

Table S2. Sequencing data and quality summary

#SampleID	Clean reads	Clean bases	GC(%)	N(%)	Q20(%)	Q30(%)
LcFGC1	22054813	6573600973	47.51	0.01	98.55	94.58
LcFGC2	23242267	6934442691	47.49	0.01	98.47	94.12
LcFGC3	21699940	6490119699	47.06	0.01	98.65	94.72
LcFGV1	23126298	6884845912	47.96	0.01	98.53	94.54
LcFGV2	23513507	7004370718	47.8	0.01	98.61	94.78
LcFGV3	23791350	7091171666	47.47	0.01	98.57	94.65
LcInC1	24611410	7354005710	48.49	0.01	98.62	94.65
LcInC2	23901142	7111166358	47.94	0.01	98.59	94.78
LcInC3	23328109	6955787706	47.69	0.01	98.62	94.85
LcInV1	22385141	6673016492	47.67	0.01	98.61	94.78
LcInV2	23338529	6965816930	47.64	0.01	98.66	94.89
LcInV3	23283685	6938198582	48.15	0.01	98.58	94.69
LcVGC1	22291616	6643658977	47.27	0.01	98.53	94.64
LcVGC2	24071886	7178005864	47.22	0.01	98.56	94.73
LcVGC3	23798799	7119965571	47.02	0.01	98.52	94.37
LcVGV1	23379951	6974124800	47.48	0.01	98.5	94.49
LcVGV2	21958325	6550406616	47.31	0.01	98.56	94.57
LcVGV3	23062818	6878662346	47.41	0.01	98.63	94.85

Table S3. Upregulated and downregulated protein profiles in gills after *Fc* immersion.

Key	log2FC(Vaccine-Control)	p_value
60S ribosomal protein L11	9.048466395257870	3.47304259725597E-23
Radixin	6.933982610642210	8.91176462876744E-25
Catalase	6.67109869228619	3.38584975353163E-18
Ig heavy chain	5.924023331048820	6.84223083123475E-18
Histone H2AX	5.794090868057400	3.9718588765506E-18
Ig heavy chain	5.493458249037830	7.30431453969535E-24
Surfeit locus protein 4	5.449623984657220	8.53920294928051E-22
Ig heavy chain	4.875143174720890	3.49855813799731E-19
Cathepsin D	4.761525119752000	3.9840683884479E-15
Annexin A2	4.350025164323390	8.40921089421165E-22
Complement component C9	4.348241668494850	1.6233955377211E-18
Microsomal triglyceride transfer protein	4.277888651474230	5.19898325474813E-24
Cathepsin B	3.825082353314320	6.84141746656184E-21
Actin, alpha cardiac muscle 1	3.61722569847705	1.6182113904359E-18
Elongation factor 2	3.433224427849860	2.94057902241842E-15
Ig heavy chain	3.0902170237624100	4.07511262417959E-17
Proteasome activator complex subunit 3	3.0697925745526000	2.31750634412687E-15
Sarcalumenin	2.946344252003290	8.81322336116624E-14
Tubulin alpha chain	2.7649678804661800	6.5539535601478E-20
ATP synthase subunit alpha, mitochondrial	2.747417137371750	2.89318402279367E-19
Pyruvate kinase PKM	2.5737353872046900	5.6988707158337E-22
ATP synthase subunit beta, mitochondrial	2.460863676877970	4.79523114991831E-19
Decorin	2.3434659007688600	1.09057283974737E-13
Tubulin beta chain	2.1063859925052300	1.51577115067586E-17

Ig heavy chain	2.0534069083763600	7.94644327009713E-17
60S ribosomal protein L18a	1.9798549841920200	2.2576076564248E-12
60S ribosomal protein L7a	1.7349501749206600	6.24106805575239E-19
Tapasin	1.7005592346503200	4.3057708044309E-19
40S ribosomal protein S24	1.4530738501680400	1.95976306155359E-16
Cell division control protein 42 homolog	1.0854404144596400	1.05981130424977E-14
40S ribosomal protein S15a	0.9656514867544990	2.19924385575779E-18
Prosaposin	0.9171300328453140	5.98296434504441E-12
Histone H2A.Z	0.873338044318654	2.52944882383038E-14
Regucalcin	0.5811830460449700	6.21799954277797E-16
Y+L amino acid transporter 2	0.03463617419096470	1.7049075892993E-05
Acrosin	-0.42234422873234900	8.88510605582422E-11
Protein NipSnap homolog 2	-0.47793180951850500	1.7265826322857E-13
40S ribosomal protein S23	-1.2392199344183400	2.08115054052698E-16
Thioredoxin	-1.24609930077278	7.79925581128135E-16
Annexin A5	-1.291357414642470	1.5953879895042E-11
Cathepsin K	-1.566493083635750	1.2791647319417E-17
Keratin, type I cytoskeletal 18	-1.9017687755506100	1.86680826924996E-18
Tropomyosin beta chain	-2.2188057777997900	7.73615572468576E-11
Hemopexin	-2.2906385222608500	6.09458533922709E-16
Fibrinogen gamma chain	-2.712780597634830	2.81649119192882E-24
Gelsolin	-2.767653620381500	2.60775941101826E-21
40S ribosomal protein S3a	-2.9805721444765600	2.18119598304468E-13
Fibronectin	-3.903272987889770	1.00186539139098E-14
Keratin, type I cytoskeletal 13	-5.026880097710760	6.35948006918134E-23
Transferrin receptor protein 1	-5.941775414035230	2.60015110703159E-21
Keratin, type II cytoskeletal 8	-6.7340295593986400	1.7661565909182E-16
Vinculin	-6.858215470317720	5.24399085887363E-14

Table S4. Upregulated and downregulated protein profiles in gills after *Vh*/*Vv*/*Pd* immersion.

Key	log2FC(Vaccine-Control)	p_value
Ig heavy chain	8.603560802759490	8.59626778450207E-28
Heat shock protein HSP 90-alpha	7.981945646026930	6.30268266259029E-20
Actin, cytoplasmic 2	7.645826766799300	2.22907439356675E-18
Ig heavy chain	7.414384192789060	5.00212612689441E-21
Actin, cytoplasmic 1	7.337439509612930	2.55868134437872E-23
Guanine nucleotide-binding protein G(t) subunit alpha	6.584895252582320	8.57036227207118E-24
Retinol dehydrogenase 10-B	6.428750833305680	3.04008923503843E-16
Proteasome subunit alpha type-7	5.959595228540000	3.51895943298715E-22
ERO1-like protein alpha	5.596370836056600	3.05113655895184E-25
Lamin-A	5.470429754740690	7.73409729409558E-22
Guanine nucleotide-binding protein G(i) subunit alpha-1	5.382978266435660	3.01513326770932E-23
Guanine nucleotide-binding protein G(i) subunit alpha-2	5.380627943664450	1.19341978467072E-25
Elongation factor 1-alpha	5.351146232458220	7.6711817509361E-21
Tropomyosin beta chain	5.236196677531440	1.88954552884502E-17
Guanine nucleotide-binding protein subunit beta-2-like 1	5.203345019119420	1.12700911508869E-21
V-type proton ATPase subunit d 1	5.026388966658110	4.36238197493875E-15
Ig heavy chain	4.807105821497570	2.12398584501543E-14
Adenosylhomocysteinase A	4.730181816606030	1.9898619464933E-25
Microsomal triglyceride transfer protein	4.72215132507371	1.31292792043156E-18
Heat shock cognate 70 kDa protein	4.645376162580290	1.35405103345402E-19

Golgi apparatus protein 1	4.597645416323930	7.26981441777298E-18
Histone H2AX	4.577251035729270	2.44261908845335E-19
Histone H2A.V	4.568886869711450	2.32461021883963E-22
Heat shock protein HSP 90-beta	4.552866386753310	4.70705159071347E-16
Leiomodin-2	4.548166709038580	2.36202881129773E-20
Alpha-actinin-4	4.522878474109450	2.912480928622E-15
Histone H2A.Z	4.51519070238367	7.72411467109302E-21
Glutathione S-transferase LANCL1	4.462435931465740	5.97438488380576E-16
AP-2 complex subunit mu-A	4.403015493749530	2.48409522214831E-23
Sarcalumenin	4.35675466756631	1.91751204664678E-26
Catalase	4.341530212318460	7.28502791051475E-22
GTP-binding nuclear protein Ran	4.318608731004760	1.52102585711451E-19
Galectin-3-binding protein B	4.266238217588790	1.88242525388264E-18
Tetraspanin-9	4.129203567001670	5.72757385704321E-22
Actin, alpha skeletal muscle B	4.1129445894820300	1.6986786865931E-19
Actin, alpha cardiac muscle	4.098174021458840	3.05624410658087E-14
Actin, alpha skeletal muscle A	4.097689049654030	2.57389567260418E-21
Alpha-actinin-1	4.0924582670077600	2.40940267246822E-19
Histone H2A	4.083752114033600	1.27066030161585E-24
Keratin, type I cytoskeletal 13	4.035595491617320	1.81198898609067E-16
Diacylglycerol O-acyltransferase 2	3.988354136714810	6.53039119069549E-14
Cathepsin D	3.9614206463235100	2.02052960459183E-15
Actin, alpha cardiac muscle 1	3.914751533190780	1.26662622445736E-24
Ig heavy chain	3.905714932274240	9.3797095153601E-13
Creatine kinase, testis isozyme	3.7630713833658900	1.7559727652319E-22
Furin-1	3.7596783614602600	5.85721870658934E-18
Plastin-2	3.747401235798770	2.99995244571135E-21
Fatty acid-binding protein 2, liver	3.740047149066270	1.19959212485407E-12
Cofilin-2	3.7213835648973700	3.01396240605529E-14

Apolipoprotein A-I	3.67300213445219	4.19695047600188E-22
Actin-related protein 2-A	3.6557980165457900	1.94292499153279E-16
Actin-related protein 2-B	3.6557889221255600	1.29161663856805E-16
Plasma membrane calcium-transporting ATPase 1	3.6448589423842900	7.05458626779429E-17
Myosin-9	3.6434698070992200	2.75814068409852E-15
Elongation factor 1-gamma	3.6236432940946600	7.80428003614117E-15
Nucleoside diphosphate kinase A2	3.612727524087400	1.99770328508365E-21
Calcium-binding mitochondrial carrier protein SCaMC-1-A	3.6123922493891000	1.92170617242301E-21
40S ribosomal protein SA	3.5339720654803000	1.55148458202406E-21
Sodium/potassium-transporting ATPase subunit alpha-1	3.426112674740770	3.91231328828546E-18
Collagen alpha-2(I) chain	3.4040654019219100	1.0724513087109E-11
Solute carrier family 15 member 2	3.401674662370350	5.01520834122471E-21
Ig heavy chain	3.341371084276740	2.30984776044642E-13
Annexin A2	3.340392567566060	5.07265637172485E-17
Hemopexin	3.3358276068503700	8.28813471158715E-22
Cell division control protein 42 homolog	3.3024692216691000	9.01516190756823E-19
Decorin	3.3000047414112200	1.40696488712878E-21
Kidney mitochondrial carrier protein 1	3.2862422481783300	1.49946901745908E-17
Ig heavy chain	3.259554981138810	1.3278062991595E-20
Y+L amino acid transporter 2	3.2574466816898800	5.40376552732104E-18
Creatine kinase M-type	3.2344081355726300	1.08184186140153E-16
Ig heavy chain	3.2118949980871600	5.75338043889077E-17
Glutathione S-transferase 3	3.187225145198400	1.75089246256783E-22
Glucosamine-6-phosphate isomerase 2	3.1282882409149800	2.0000460406708E-20
Myosin-11	3.1104372838451300	2.42631644373289E-12
Ig heavy chain	3.0923445445581900	9.72639210378949E-22
Glyceraldehyde-3-phosphate dehydrogenase	3.0450991880827700	2.84489055877976E-14
Peroxiredoxin-1	3.0238733498316000	1.68016248618673E-16

Ig heavy chain	3.014797989856500	2.19632648280516E-22
Ubiquitin-40S ribosomal protein S27a	3.0056349927545300	2.32724049567963E-21
Surfeit locus protein 4	2.9933720849971900	7.89739053050708E-20
Tubulin alpha chain	2.977423074430420	2.44004872116191E-18
NADP-dependent malic enzyme	2.963594399791240	1.26871472940903E-12
Polyubiquitin-B	2.9610023734942700	7.50347629854726E-22
Spliceosome RNA helicase DDX39B	2.919099944805240	3.62322696057757E-19
Reticulon-1-A	2.866756795296480	4.59156063589323E-14
Ig heavy chain	2.826607550829590	1.39274404911153E-21
Solute carrier family 12 member 2	2.8185240588702700	3.52594177691283E-18
Tubulin beta chain	2.8175670012475700	8.5525053265414E-20
Neoverrucotoxin subunit alpha	2.7638297861689800	1.35006355667423E-23
Cysteine and glycine-rich protein 1	2.7147137572300400	2.72879755629294E-18
Elongation factor 2	2.696811647622640	1.00522133325462E-16
Ras-related protein Rab-10	2.6500480090391500	6.79158609773263E-15
Fibrinogen beta chain (Fragment)	2.6361938413147100	1.3108966991948E-21
Ig heavy chain	2.630110034343380	2.47777675960821E-12
Gelsolin	2.6071065262345200	1.06321004657222E-18
60S ribosomal protein L15	2.6024443855286600	1.24553263123024E-21
Actin-related protein 2/3 complex subunit 4	2.598168752023050	5.87842221505294E-20
Eukaryotic initiation factor 4A-II	2.597570564248240	8.16356693177352E-13
Hemoglobin subunit beta-A	2.553466549166600	1.9339406052531E-12
40S ribosomal protein S7	2.548688543516460	1.02347448405359E-14
Tubulin beta-1 chain	2.5292191819521000	3.51265579977865E-19
Unconventional myosin-Ic	2.501979331348020	7.8012024530106E-15
Saxiphilin	2.488794459345790	7.24457698746198E-13
Proteasome activator complex subunit 3	2.463463078634670	6.03862485499293E-26
Integrin beta-1	2.4547170199532200	2.38101231956579E-25
Cytochrome P450 3A40	2.4426562489224900	2.71114207706561E-21

Keratin, type II cytoskeletal 8	2.424859211729620	1.14374199885411E-20
Ubiquitin-60S ribosomal protein L40	2.4235756370219000	2.28346224639832E-17
Amine oxidase [flavin-containing]	2.410145992747080	4.78447888350658E-23
Xanthine dehydrogenase/oxidase	2.3765113148935200	7.68298735710622E-19
Seizure protein 6 homolog	2.3488771035531100	1.23599262348113E-17
ATP synthase subunit alpha, mitochondrial	2.32175867421482	1.06559243381324E-15
Arrestin red cell isoform 3	2.312225339502740	1.49464521072478E-21
Galectin-3-binding protein A	2.299877185310280	1.18959598977602E-11
Disintegrin and metalloproteinase domain-containing protein 10	2.2938823588770200	1.67721977853009E-18
Unconventional myosin-VI	2.286696329094700	2.98774858085585E-19
Angiotensin-converting enzyme (Fragment)	2.2758600538270200	6.06401461129718E-12
40S ribosomal protein S25	2.2693471529144700	3.74009805546824E-18
Collagen alpha-3(VI) chain	2.1483662777632600	1.47270561854902E-17
F-actin-capping protein subunit alpha-2	2.140866706702840	2.88051419036239E-18
Ras-related protein Rab-8	2.0961293705998600	2.10857741838105E-21
Pyruvate kinase PKM	2.074052209476260	6.15543496566326E-14
Ras-related protein Rab-8A	2.067634658019720	4.44111963966949E-23
Aspartate aminotransferase, mitochondrial	2.0339308313539100	4.64288447597664E-15
Collagen alpha-2(VI) chain	2.0088851871612400	2.68746087798784E-16
Caspase-3	1.9773089048727600	1.920224746914E-18
40S ribosomal protein S3a	1.9766208393472300	8.77388500626277E-17
Venom phosphodiesterase 1	1.9410848188718500	5.10579798143877E-20
Rho-related GTP-binding protein RhoA-C	1.9269314444068500	5.58980926024033E-21
Rho-related GTP-binding protein RhoA-D	1.9269306739557200	1.36531053776933E-22
Rho-related GTP-binding protein RhoA-B	1.9269300978496100	1.82989459608862E-22
T-complex protein 1 subunit delta	1.91949187960099	1.02113161266878E-12
WW domain-containing oxidoreductase	1.9152935763038200	5.94958206108814E-19
Cytosolic 10-formyltetrahydrofolate dehydrogenase	1.9146264032723600	5.69490125211316E-20

ATP synthase subunit beta, mitochondrial	1.9109407228754000	2.87956967044144E-19
Tetraspanin-33	1.8705352586516200	2.81207962960633E-13
Radixin	1.8405491538683000	2.20293469462857E-13
Ig heavy chain	1.8260478534571700	6.14687593096984E-20
Aldo-keto reductase family 1 member A1	1.7910409987502000	2.39403462127038E-12
Fermitin family homolog 2	1.7687712135501600	3.62083978212027E-13
Serotransferrin	1.7381588437808500	9.08325545247095E-21
Adenosylhomocysteinase B	1.6964147201218400	4.51633693109869E-16
Glyceraldehyde-3-phosphate dehydrogenase 2	1.6289758013350900	2.73874318406267E-19
Unconventional myosin-Va	1.622778938744310	8.2789779617738E-14
Cytosol aminopeptidase	1.5846014641840800	2.17209511381121E-14
Fibronectin	1.5795762142529600	6.50833294165015E-19
Guanine nucleotide-binding protein G(I)/G(S)/G(T) subunit beta-1	1.5640564057324700	7.87274657570657E-20
Ig heavy chain	1.5209691936402100	1.89586817179895E-14
Histone H2B 1/2	1.5162151153390300	2.26297180233463E-18
Acrosin	1.4771199199068100	1.63633800361817E-18
Urokinase-type plasminogen activator	1.4771091442226800	4.334392431578E-15
40S ribosomal protein S3	1.471642547326080	2.33260510811274E-21
Mucin-5B	1.4376671576297500	1.13511855847767E-20
Cytochrome b-c1 complex subunit Rieske, mitochondrial	1.4242722648913500	7.9445005861088E-21
Alpha-N-acetylgalactosaminidase	1.4187399663801500	4.88098835614704E-21
Collagen alpha-1(I) chain	1.4016749412933000	4.68194533186907E-18
Sarcoplasmic/endoplasmic reticulum calcium ATPase 2	1.375204835141990	3.87166923474858E-14
Netrin-1	1.3611683329908300	1.61182288879249E-12
Laminin subunit beta-4	1.3508966774462500	2.32258901600469E-19
Cornifelin homolog	1.3450718900186000	1.73943766659065E-16
Single-stranded DNA-binding protein 1-A, mitochondrial	1.337244642975150	7.82168483668412E-15

Band 3 anion exchange protein	1.3253861434646600	3.66534833242503E-14
Low choriolytic enzyme	1.3212505183280200	7.64335502364028E-16
Superoxide dismutase [Cu-Zn]	1.3180473094465000	1.60148940882767E-12
60S ribosomal protein L18a	1.3124168055276000	1.54483758270165E-17
Choline transporter-like protein 1	1.300748265029740	8.09286975083903E-20
Ras-related protein Rab-2A	1.2941408684405800	4.41574067586359E-17
Dihydrolipoyllysine-residue succinyltransferase component of 2-oxoglutarate dehydrogenase complex, mitochondrial (Fragment)	1.282222574832400	2.03861157685777E-16
Peptidyl-prolyl cis-trans isomerase B	1.2015084516022500	3.83452217322358E-17
60S ribosomal protein L7a	1.1862781778280900	1.26454406055802E-12
Sarcoplasmic/endoplasmic reticulum calcium ATPase 3	1.1800735770591100	1.82249099505912E-18
Alpha-actinin-2	1.1716549784065800	2.2241905353974E-10
Glutamate dehydrogenase, mitochondrial	1.1672961062724500	5.23344039199659E-17
60S ribosomal protein L35a	1.1400965007664800	1.11163700630993E-20
Transferrin receptor protein 1	1.1388632582682200	4.80198880011849E-17
Trypsin-2 (Fragment)	1.1256537952284800	3.76074064287282E-17
Collagen alpha-1(VI) chain	1.091334041621190	9.4155774084596E-13
Solute carrier family 25 member 33	1.0693943770930400	6.01888461983025E-11
Epidermal growth factor-like protein 6	1.0571600805502800	3.20031511337249E-16
Coatomer subunit beta	1.050343059249740	2.31029893658251E-19
Aspartate aminotransferase, cytoplasmic	1.0118759428114700	6.26889648286685E-14
Prosaposin	1.0002126585400100	1.62530707613069E-12
Alpha-tectorin	0.959886995588203	5.73821813427876E-13
Trypsin II-P29	0.942635415997767	3.28108302320977E-11
Annexin A5	0.9307918633348570	1.02137165432437E-15
Histone H4	0.8939789286962320	1.55775943812795E-18
Ras-related protein Rab-11B	0.8800886948251580	1.49861830207346E-19
Malate dehydrogenase, cytoplasmic	0.8487275048684500	3.14422297896818E-13

Sarcoplasmic/endoplasmic reticulum calcium ATPase 1	0.8151903164418140	5.04662733692198E-19
Laminin subunit gamma-1	0.760957097536032	3.48136718696988E-15
Serine/threonine-protein phosphatase 2A catalytic subunit alpha isoform	0.6840408035000430	3.94266396806352E-18
Tapasin	0.638561400211497	3.60926238188906E-10
Ladderlectin	0.5789432405734840	3.16473347890165E-13
Cathepsin K	0.5094269146918830	2.54111973675011E-15
Ferritin, middle subunit	0.5011790285599740	2.22239353938873E-14
UPF0462 protein C4orf33 homolog	0.4230188663265690	3.34684225999097E-08
Phosphotriesterase-related protein	0.3578230911620470	7.28675383191639E-12
Vinculin	0.35380920741361900	2.73264791081492E-10
Complement component C9	0.3148814202982850	4.343370997941E-10
Triosephosphate isomerase B	0.29997251189014300	6.07833598030741E-10
L-rhamnose-binding lectin SML	0.17222170479222900	3.23202542869993E-12
Collagen alpha-1(XII) chain	0.1568787424074000	1.8659675288511E-08
Actin-related protein 3-A	0.0783057985685609	3.2048590377958E-06
Choline transporter-like protein 2	-0.08548828465396950	1.52351533887969E-08
Glycerophosphocholine cholinephosphodiesterase ENPP6	-0.2541459024218080	3.93247230168257E-15
Procathepsin L (Fragments)	-0.284309111196432	1.1300193369628E-09
Beta-enolase	-0.2981148559529420	2.0554966073095E-10
Complement C3 (Fragment)	-0.3017673378313750	5.31572498869518E-15
Mediator of RNA polymerase II transcription subunit 24	-0.31853539385963700	4.14454267907194E-10
Alcohol dehydrogenase 1	-0.33455018781786100	9.78396778700422E-11
60S ribosomal protein L11	-0.3704931483286980	6.52225894311033E-13
Isochorismatase domain-containing protein 2	-0.42575765019540100	6.88854310924904E-14
Leukocyte elastase inhibitor	-0.4336585670047020	2.36955094828778E-15
Thioredoxin	-0.4695191907815720	2.46311556913395E-14

Low-density lipoprotein receptor-related protein 1	-0.5201946881938650	2.61259968309876E-12
High choriolytic enzyme 1	-0.5655090476121710	1.52065468331546E-16
Phospholipase-B 81	-0.5677222433616010	1.39616140453102E-12
Fibrinogen gamma chain	-0.6543696278608630	1.46658050500793E-19
Alpha-enolase	-1.0707984632378800	2.95128353086002E-11
Hydroxysteroid dehydrogenase-like protein 2	-1.080944684441540	1.84504804989478E-15
Serine/threonine-protein phosphatase CPPED1	-1.1017761199869200	2.97818452119778E-17
Cystatin-B	-1.3671932897419700	2.9477574404006E-19
Alpha-1-antitrypsin homolog	-1.5271208795322400	5.84678384208276E-16
Trypsin-3 (Fragment)	-1.5609418701429700	3.57713393604255E-23
Retinol-binding protein 4-A	-2.2140936406442800	1.23959252730265E-19
Carboxypeptidase O	-2.6761744299180200	1.81845112173003E-13
Chymotrypsin B	-3.350314973254330	9.32999484812915E-18
Elastase-1	-3.4223680602582000	1.28147507610466E-17
Keratin, type I cytoskeletal 18	-3.5100869671534200	1.16013859057268E-22
Neoverrucotoxin subunit beta	-3.810924457526580	5.4057029319925E-20
Venom factor	-3.988230434474480	1.4903862406878E-21
Chymotrypsin A	-4.435055057854880	5.26349859467856E-15
Acid phosphatase type 7	-6.726960737772680	3.37014098812503E-22
Cathepsin B	-7.01268465789051	2.5366312009615E-16

Table S5. Upregulated and downregulated protein profiles in intestine after *Vh*/*Vv*/*Pd* injection.

Key	log2FC(Vaccine-Control)	p_value
Ig heavy chain	10.59540792785120	1.69659665323373E-26
Ig heavy chain	8.858192152258880	1.70781738766284E-16
Biotinidase	7.54658595362962	6.11530074773315E-22

Ig heavy chain	7.202978789067660	1.86281835578462E-20
Ubiquitin-60S ribosomal protein L40	7.07603428841967	2.19132113121435E-26
Ig heavy chain	5.353707414469350	6.46423720179588E-16
Angiotensin-converting enzyme (Fragment)	5.198775424173920	2.67316863067018E-22
Elongation factor 1-alpha 1	4.85610572715715	4.78539267961545E-20
Elongation factor 1-alpha	4.856099163102220	3.04352909799917E-22
Alpha-enolase	4.762122551764840	8.38441409589824E-24
Leiomodin-2	4.294016170853620	5.05975556252139E-20
Hydroxysteroid dehydrogenase-like protein 2	4.292768004278490	5.6276471378365E-13
Beta-enolase	4.270374428648290	4.3980785127094E-13
Microsomal triglyceride transfer protein	4.209318962892400	1.04468969942659E-14
Caspase-3	4.097047686288680	8.22627557476232E-20
40S ribosomal protein S23	3.9702687156409400	1.98963305782691E-19
Ig heavy chain	3.7202530611898300	6.13132591960687E-13
Ras-related protein Rab-14	3.5757769291104600	1.93352307540285E-16
Glyceraldehyde-3-phosphate dehydrogenase	3.4819172587295100	1.00177442865626E-18
40S ribosomal protein S7	3.37889333651464	1.00155268580721E-13
Aldo-keto reductase family 1 member A1	3.358582414780700	1.5096023457374E-19
Ferritin, middle subunit	3.2267404058277300	1.02452765879285E-22
Ig heavy chain	3.178622679628130	2.19484497482506E-20
Gelsolin	3.1493697477182000	1.29207962517171E-12
60S ribosomal protein L11	3.0011172215382100	6.74988309549044E-15
Chymotrypsin B	2.9808901699703300	1.51386015492946E-17
Ig heavy chain	2.8934403012472800	7.64731045205929E-22
Ig heavy chain	2.8757745157008800	1.32407488298379E-21
Radixin	2.8389598231806600	5.53877514105066E-21
Guanine nucleotide-binding protein G(I)/G(S)/G(T) subunit beta-1	2.773641361214290	5.49345744249777E-22
Glucosamine-6-phosphate isomerase 2	2.721626037574010	9.48748424649424E-13
Phospholipase-B 81	2.6909342711417800	2.48274885482467E-21

Eukaryotic initiation factor 4A-II	2.670528504342590	2.24458217830763E-19
Acid phosphatase type 7	2.653091800811130	4.44034415174432E-17
Ras-related protein Rap-1b	2.6151180068849900	2.37237894402147E-23
Ras-related protein ORAB-1	2.5741461437059600	1.39957597089583E-21
Ras-related protein O-Krev	2.556522230289040	5.39863678864632E-19
Trypsin-3 (Fragment)	2.5332332730904300	1.58381225121156E-17
Adenosylhomocysteinase A	2.5006117355641700	2.280326222928E-11
Diacylglycerol O-acyltransferase 2	2.4869327023518900	1.24101266397888E-22
Aspartate aminotransferase, mitochondrial	2.4569889293377600	4.431420469781E-13
Peroxiredoxin-1	2.3816572363144900	2.2390649618255E-15
Tapasin	2.3387659409521000	3.56920354660059E-14
AP-2 complex subunit mu-A	2.335120736097140	6.38656061079426E-17
Acyl-CoA-binding protein homolog	2.313789959235930	5.19329803030577E-25
Fibrinogen gamma chain	2.274206747192490	2.94370878307172E-14
40S ribosomal protein S13	2.2718896581885800	8.681190403847E-21
Actin, cytoplasmic 1	2.2619775024523800	2.67867204764985E-12
Carboxypeptidase O	2.251602695443190	3.68706645030581E-17
Annexin A2	2.2300236486696400	1.08741027377888E-14
Plasma membrane calcium-transporting ATPase 1	2.201865167538750	6.73030057951518E-17
Creatine kinase M-type	2.170429778882450	6.76281912692118E-18
Proteasome subunit alpha type-7	2.1264097356346100	4.24017856624804E-20
60S ribosomal protein L7a	2.102032849214510	1.44727648324989E-18
ATP synthase subunit alpha, mitochondrial	1.9899319942679200	4.7849631425295E-13
Actin, cytoplasmic 2	1.9547354144227500	7.17464023890223E-14
Thioredoxin	1.9363650957811100	3.71480241002893E-23
Ig heavy chain	1.9157079214113300	1.42062462677315E-11
Heat shock protein HSP 90-beta	1.8997105232589900	6.57805336448682E-13
Heat shock protein HSP 90-alpha	1.8993250969970100	7.95389837013053E-21
Transferrin receptor protein 1	1.8894461037798000	3.14744994172184E-11

Aspartate aminotransferase, cytoplasmic	1.8538901405144000	6.03674872461409E-16
Trypsin II-P29	1.8470026141504300	1.17078836197584E-16
Actin, alpha cardiac muscle 1	1.8313864736794400	7.14813741958153E-17
Adenosylhomocysteinase B	1.8247530308722200	1.40271658502323E-18
Protein ABHD14A	1.8214201790326800	2.01479697673667E-16
Histone H2B 1/2	1.7998768468554100	1.19350302377525E-19
Ras-related protein Rab-11B	1.7361375411261200	4.48403292370118E-19
Fermitin family homolog 2	1.7311387246207900	1.08006474356024E-16
ATP synthase subunit beta, mitochondrial	1.655809000887580	1.13656205156492E-18
NADP-dependent malic enzyme	1.6375015645209000	5.19717999764024E-14
Trimethyllysine dioxygenase, mitochondrial	1.6292362436739200	3.02230961364321E-15
Calcium-binding mitochondrial carrier protein SCaMC-1-A	1.6177642788335400	5.04837680167255E-18
Triosephosphate isomerase B	1.597471246207530	8.24507377838036E-18
Cathepsin K	1.5579866694184300	8.23329674553442E-12
Cathepsin D	1.5436180364369400	6.50707032839341E-16
Hemopexin	1.5311533941730700	8.39034233405319E-12
Retinol dehydrogenase 10-B	1.5224208144862700	7.1744667193133E-16
60S ribosomal protein L10	1.5194105400214800	1.31195896665595E-19
Serine/threonine-protein phosphatase 2A catalytic subunit alpha isoform	1.5166736198433700	6.16641442917416E-14
Tetraspanin-31	1.4958333629359400	2.52325973590578E-17
Actin, alpha skeletal muscle B	1.4884988758706600	2.00576287457264E-12
Alpha-actinin-2	1.4829080099147200	4.94546205744418E-15
Actin, alpha cardiac muscle	1.4714816781145200	4.37464983607882E-13
Actin, alpha skeletal muscle A	1.4278088225800000	3.65591759440902E-15
High choriolytic enzyme 1	1.4171252335997700	8.61440560884946E-11
Malate dehydrogenase, cytoplasmic	1.4106830313723900	7.54338096633486E-14
Hemoglobin subunit alpha-A	1.405708461981210	9.0227924541258E-15

Dihydrolipoyllysine-residue succinyltransferase component of 2-oxoglutarate dehydrogenase complex, mitochondrial (Fragment)	1.3702461911511500	1.37851913589584E-19
Actin-related protein 2/3 complex subunit 4	1.365129741802560	9.49640149288614E-15
Guanine nucleotide-binding protein G(t) subunit alpha	1.3646321538362900	3.82181241573849E-11
Ras-related protein Rab-8A	1.346226220306160	6.61146296010186E-12
Tubulin beta-1 chain	1.3373520551306600	7.655779254196E-19
Sarcalumenin	1.3333732202386200	1.76998118198722E-13
Low choriolytic enzyme	1.3190181756553000	2.90658838332836E-19
Catalase	1.3158185152316900	1.72438635230413E-18
Ras-related protein Rab-2A	1.3099603479779400	7.8780975561806E-20
NADPHcytochrome P450 reductase (Fragments)	1.2931293544783500	1.22002728035619E-18
Histone H4	1.289291909942450	8.874675702427E-23
40S ribosomal protein SA	1.2854885654946500	6.56769054485695E-19
Unconventional myosin-VI	1.2813809915440200	1.97122146179247E-16
Cytosolic 10-formyltetrahydrofolate dehydrogenase	1.2776535433820400	3.40774208962925E-10
Guanine nucleotide-binding protein G(i) subunit alpha-1	1.2758326910326500	1.89272195768323E-17
Ig heavy chain	1.2695239497313500	1.40111517870248E-17
Ras-related protein Rab-8	1.2656069694152600	1.28282610526544E-13
Guanine nucleotide-binding protein G(i) subunit alpha-2	1.2554966148064500	3.74411421907225E-22
T-complex protein 1 subunit delta	1.247195164689320	8.41331671916303E-13
Keratin, type I cytoskeletal 18	1.1589333964905700	1.11216983053045E-16
UPF0462 protein C4orf33 homolog	1.1136238769310700	2.20398246503225E-10
Keratin, type I cytoskeletal 13	1.1071469901719200	2.52705545873106E-14
Membrane-associated progesterone receptor component 1	1.1025841142233700	1.65241766572566E-17
Amine oxidase [flavin-containing]	1.1018303659454200	5.01168704549739E-13
Hemoglobin subunit beta-A	1.084333027795380	8.10377378862829E-15

Arrestin red cell isoform 3	1.0694175714654300	1.7565982736195E-12
Histone H2A	1.0608617401352900	2.23386713590592E-18
Trypsin-2 (Fragment)	1.0575197796037300	4.76088898149725E-14
Serine/threonine-protein phosphatase CPPED1	1.0564941574330700	2.1243288317849E-15
Alpha-1-antitrypsin homolog	1.0337987104313200	8.91715734585559E-19
Laminin subunit beta-4	1.0283192592410200	4.94759318281792E-16
Collagen alpha-1(XII) chain	1.0050312809332100	2.25496561366153E-14
Cytochrome P450 3A40	0.9964241573860700	1.17189596871313E-18
Plasma membrane calcium-transporting ATPase 2 (Fragment)	0.995673792202286	1.09451279921662E-11
Cofilin-2	0.9892121968674280	4.40506990717662E-11
Prosaposin	0.9831818691414150	1.46271154923018E-16
Isochorismatase domain-containing protein 2	0.9705043765012750	1.80720268689439E-09
Plastin-2	0.9662512130658850	1.37289328721698E-22
Galectin-3-binding protein A	0.9657847129164380	4.15303435659385E-18
Stonustoxin subunit alpha	0.9502711755416440	4.30737509260063E-12
Surfeit locus protein 4	0.9469379182967690	4.63512591995566E-13
Solute carrier family 15 member 2	0.9446167105504130	3.80796223404923E-14
Fibrinogen beta chain (Fragment)	0.9258654131641140	2.03948407557579E-10
Tubulin beta chain	0.8999469030017090	1.64714604045204E-20
60S ribosomal protein L35a	0.8979520191187550	1.04425827335669E-20
Alpha-actinin-4	0.8793917586429990	2.45868916874804E-18
Sarcoplasmic/endoplasmic reticulum calcium ATPase 2	0.8669048698651520	1.70215624290681E-23
Sarcoplasmic/endoplasmic reticulum calcium ATPase 1	0.8575259490017720	3.44064921305507E-12
Pyruvate kinase PKM	0.834176787712714	4.52791903740308E-13
Protein NipSnap homolog 2	0.8335246167637040	2.85065365226106E-16
Sarcoplasmic/endoplasmic reticulum calcium ATPase 3	0.8312507252075290	3.57614332524043E-11

Cytochrome b-c1 complex subunit Rieske, mitochondrial	0.8226503646006440	1.5358763029239E-18
Mediator of RNA polymerase II transcription subunit 24	0.8131496554757690	4.54885692275903E-13
Coatomer subunit beta	0.8116752423567170	3.1364404639506E-15
Laminin subunit gamma-1	0.8090180452513990	7.16020496530351E-14
Cornifelin homolog	0.7952589797105000	5.34033765390602E-18
Apolipoprotein A-I	0.7907830004583620	6.46750756746927E-16
Alcohol dehydrogenase 1	0.7727592685120080	1.66288821661388E-14
Neoverrucotoxin subunit beta	0.7705441932983630	1.5831234351255E-18
Proteasome activator complex subunit 3	0.76059558502606	1.75998763213277E-17
Keratin, type II cytoskeletal 8	0.7547214782829460	1.00599190967112E-13
Myosin-11	0.7449155543263930	2.52069166735668E-11
Histone H2A.Z	0.733149723520377	1.5838092351978E-17
Histone H2A.V	0.7331474568546560	2.14745125514359E-17
Complement C3 (Fragment)	0.7326378586603400	1.15400818961776E-10
F-actin-capping protein subunit alpha-2	0.721068071778479	4.94232963790362E-13
Vinculin	0.7051035258167820	2.73863540181136E-14
Alpha-N-acetylgalactosaminidase	0.6967644263070730	1.79364271575788E-14
Procathepsin L (Fragments)	0.6904813174868030	4.13397350844005E-13
Histone H2AX	0.6812534607822370	1.19556601115795E-12
Reticulon-1-A	0.6463456000172660	1.23992160608231E-16
Xanthine dehydrogenase/oxidase	0.6382436355895890	1.00347290649983E-10
Actin-related protein 3-A	0.6370646101239790	1.04420127101329E-10
Glycerophosphocholine cholinephosphodiesterase ENPP6	0.6281357896525430	4.36342971460071E-13
Neoverrucotoxin subunit alpha	0.5998979930692700	1.83227130345954E-10
Tubulin alpha chain	0.5948524439773340	5.61534736665745E-18
40S ribosomal protein S3	0.5829686498378170	4.90077390122463E-17
40S ribosomal protein S24	0.5810829388039100	1.22531327094238E-15

Glutathione S-transferase 3	0.5803709675008190	1.53666243993178E-16
WW domain-containing oxidoreductase	0.5756806410063750	6.64206163727928E-12
Glyceraldehyde-3-phosphate dehydrogenase 2	0.5641482867835170	3.01080705984672E-12
Ras-related protein Rab-10	0.5517389139613190	1.57825662032583E-14
Kidney mitochondrial carrier protein 1	0.5460448225278360	2.4300752628217E-11
Actin-related protein 2-A	0.5415754281656180	1.047493093175E-10
Actin-related protein 2-B	0.5415729989283240	3.65587317808342E-12
Alpha-tectorin	0.5185399410345790	4.18203047275786E-11
Solute carrier family 12 member 2	0.5111343749586580	1.11497897448496E-17
Elongation factor 1-gamma	0.50691137740165	1.49367513707047E-14
Receptor expression-enhancing protein 6	0.4921745329856760	3.5266123944542E-15
Aminopeptidase Ey	0.4856483318772750	2.58597412638527E-13
Src substrate protein p85	0.47530742355479900	4.27725187929519E-13
Spliceosome RNA helicase DDX39B	0.46802453581114600	6.61906532537351E-16
Single-stranded DNA-binding protein 1-A, mitochondrial	0.4503871924778980	4.48177520507346E-15
Serine/threonine-protein phosphatase PP1-gamma catalytic subunit A	0.44973325529206700	2.35356590253167E-10
Urokinase-type plasminogen activator	0.4334602260461120	7.81444993614821E-17
Acrosin	0.43345497922616600	4.95564462044832E-10
ERO1-like protein alpha	0.4222959411827210	1.79105104232254E-11
Low-density lipoprotein receptor-related protein 1	0.41843680884560200	2.37053575771723E-15
Cysteine and glycine-rich protein 1	0.4110030367980930	3.35796706393034E-16
Leukocyte elastase inhibitor	0.4042192765676480	8.32299439314812E-13
Serine/threonine-protein phosphatase PP1-beta catalytic subunit	0.3961693183170180	5.15327202682011E-11
Glutathione S-transferase LANCL1	0.3824793046949790	1.6870030920559E-13
Collagen alpha-2(VI) chain	0.37975922731811800	5.48054258510262E-09
60S ribosomal protein L18a	0.36411470751782100	1.59493708802363E-14
Unconventional myosin-Ic	0.36322332734727400	1.8494573551914E-10

Phosphotriesterase-related protein	0.3510645186202060	1.90672543862592E-13
Stonustoxin subunit beta	0.3368631228738860	3.83316403222954E-09
GTP-binding nuclear protein Ran	0.3230525845506730	8.41144921848512E-14
Decorin	0.3169755409477380	2.61440634660387E-15
40S ribosomal protein S15a	0.284482379965489	1.80888771356029E-08
Elongation factor 2	0.2788017023203010	6.01288756973237E-17
Annexin A5	0.24149001909201600	2.99355862752038E-08
Tropomyosin beta chain	0.21023021918322700	5.23879890195868E-14
Tetraspanin-33	0.20096233441001800	1.85823546877011E-10
Collagen alpha-3(VI) chain	0.18323314523988900	2.5501802610877E-14
L-rhamnose-binding lectin SML	0.18255095832195400	4.2505681957833E-13
40S ribosomal protein S3a	0.1765675026977470	1.87312546313623E-13
Venom phosphodiesterase 1	0.16611585184557500	3.27515194793112E-12
Complement component C9	0.11282728207503800	4.62990560381562E-09
Phosphoenolpyruvate carboxykinase, cytosolic [GTP]	0.08658988045420560	6.07098657337399E-12
Seizure protein 6 homolog	0.08216305315569980	3.54164431864255E-11
V-type proton ATPase subunit d 1	0.06465266252102000	7.27565960403408E-10
Myosin-9	0.06408511468795550	7.03957324075661E-08
40S ribosomal protein S16	0.059258580040246800	9.29656636888648E-07
Band 3 anion exchange protein	0.019142008366234400	0.005496171915987820
Chymotrypsin A	-0.06373611728746550	1.27607256605202E-08
60S ribosomal protein L15	-0.089375850801936	2.5647798270038E-07
Y+L amino acid transporter 2	-0.09547401713660260	2.32769692685372E-06
Cystatin-B	-0.13137355270611900	1.23294891304438E-09
Protein rapunzel	-0.1534078944440050	3.50037448328466E-12
Saxiphilin	-0.1561084216351500	5.33489619832343E-10
Sodium/potassium-transporting ATPase subunit alpha-1	-0.16492837729581900	9.40392194996978E-14
Phosphoglycerate kinase	-0.1778236782801380	7.86354080804299E-11

Creatine kinase, testis isozyme	-0.2154328854989170	3.35494496433262E-11
SuccinateCoA ligase [ADP/GDP-forming] subunit alpha, mitochondrial (Fragment)	-0.23447205061884400	2.07429235233449E-10
Netrin-1	-0.2519979427428610	1.07679290380293E-14
Alpha-actinin-1	-0.25415375941824400	1.98292892327508E-13
Peptidyl-prolyl cis-trans isomerase B	-0.3355937051563030	6.22641510386555E-13
Lamin-A	-0.34436680932216000	1.7170143535097E-12
Choline transporter-like protein 1	-0.34494598948005600	3.744491291033E-13
Solute carrier family 25 member 33	-0.3549456110026410	3.5349570056941E-12
Rho-related GTP-binding protein RhoA-B	-0.394390645201387	1.60643103651881E-09
Rho-related GTP-binding protein RhoA-C	-0.394397240754941	9.70885308929834E-17
Rho-related GTP-binding protein RhoA-D	-0.3943981671539840	2.26057712221017E-14
40S ribosomal protein S25	-0.39755999000232100	2.55798423975778E-12
Cell division control protein 42 homolog	-0.40583436043614200	5.37944434241122E-13
Unconventional myosin-Va	-0.43978180756275300	8.05605474337047E-16
Fibronectin	-0.44187486110283900	1.12531970389343E-10
Mucin-5B	-0.4625750120108270	4.12401908784601E-11
Galectin-3-binding protein B	-0.476392595806054	1.46720005361074E-08
Integrin beta-1	-0.5309403578392070	2.21832280871812E-17
Elastase-1	-0.5590431591512050	4.86466153049386E-14
Disintegrin and metalloproteinase domain-containing protein 10	-0.5970447745874880	1.41058522554136E-14
Collagen alpha-2(I) chain	-0.6040896440882190	3.30425046805742E-10
Furin-1	-0.6720924908501790	1.91667174633301E-19
Superoxide dismutase [Cu-Zn]	-0.6935902336304670	1.15870406287216E-15
Serotransferrin	-0.7316245034440490	6.18396593401197E-16
60S ribosomal protein L4-B	-0.7753007655488450	1.45301204211536E-13
Fatty acid-binding protein 2, liver	-0.7835067307126100	1.09094788445579E-17
Collagen alpha-1(VI) chain	-0.8746992734967630	4.11463203625601E-12
Venom factor	-0.9214637927779000	1.07126853032612E-15

Choline transporter-like protein 2	-0.9938730787683530	4.51728625132208E-13
Cathepsin B	-1.0095221016103800	1.43464898776276E-17
Extracellular sulfatase Sulf-1	-1.2343669119950500	5.91913616462457E-14
40S ribosomal protein S17	-1.2573425263014600	2.346625885296E-16
Peroxisomal bifunctional enzyme	-1.2943604298194900	1.3578100710246E-09
V-type proton ATPase subunit B	-1.4533563686736700	4.03287850660359E-10
Ladderlectin	-1.4826972285118700	3.57579972631632E-20
Epidermal growth factor-like protein 6	-1.5292325174896300	2.32260166722318E-15
Endoplasmic reticulum chaperone BiP	-1.6805014627698400	4.2776533052649E-14
Collagen alpha-1(I) chain	-1.6934405126134100	2.480760116298E-21
Heat shock cognate 70 kDa protein	-1.8000234362887100	2.84988277047096E-13
Golgi apparatus protein 1	-2.01250967989208	3.09927706946126E-11
Tetraspanin-9	-2.3179761443787900	4.44215999044935E-16
40S ribosomal protein S18	-2.61222603518355	2.986425285214E-20
